# Reconsidering music in stroke rehabilitation: a scoping review from auditory stimulus to relational process

**DOI:** 10.3389/fpsyg.2026.1774971

**Published:** 2026-06-05

**Authors:** Soo Ji Kim, Bumsuk Ko, Hyunjung Lee, Kyurim Kang, Heejin Lee, Yoo Jeong Kim

**Affiliations:** 1 Music Therapy Education, Graduate School of Education, Ewha Womans University, Seoul, Republic of Korea; 2 Department of Music, Graduate School, Hansei University, Gunpo, Republic of Korea; 3 Graduate School of Professional Therapy, Gachon University, Seongnam, Republic of Korea; 4 Center for Music and Medicine, Department of Neurology, Johns Hopkins University School of Medicine, Baltimore, MD, United States; 5 Graduate School of Music Therapy, Sookmyung Women’s University, Seoul, Republic of Korea; 6 Department of Music Therapy, Graduate School, Ewha Womans University, Seoul, Republic of Korea

**Keywords:** motor rehabilitation, music-based intervention, scoping review, sound, stroke

## Abstract

**Introduction:**

Over the past three decades, music has been increasingly incorporated into stroke motor rehabilitation; however, the term *music-based intervention* has been applied inconsistently. Interventions range from simple rhythmic cues to complex interactive activities, yet these distinctions are often insufficiently described to allow meaningful comparison across studies.

**Methods:**

This scoping review examined how music and sound have been conceptualized and applied in stroke motor rehabilitation research published between 1993 and 2023. Ninety-seven studies were identified through major databases. Data were extracted on definitions of music and sound, auditory stimulus characteristics, delivery methods, and provider expertise, followed by numerical and thematic analyses.

**Results:**

Substantial heterogeneity was found in how musical elements and auditory designs were reported, with many studies lacking essential information on stimulus structure. Comparative analysis identified three overarching approaches: (1) stimulus-based methods targeting movement timing, (2) task-based methods involving rhythmic or instrumental performance, and (3) process-based methods emphasizing relational and interactive engagement. These approaches were positioned along a continuum ranging from mechanically oriented to relationship-centered interventions.

**Discussion:**

The findings highlight persistent conceptual ambiguity between music and sound and underscore the need for clearer and more systematic reporting of musical parameters. Conceptualizing music as a multidimensional therapeutic component may support stronger integration of neuroscientific and clinical perspectives when explaining mechanisms of stroke motor recovery.

## 1 Introduction

As the neuroscientific principles of music are increasingly studied, interest in the therapeutic application of music within neurorehabilitation has grown. Research has revealed how music and rhythm are processed across different regions of the brain (Särkämö et al., 2008; Lezama-Espinosa and Hernandez-Montiel, 2020), providing evidence that music can influence motor, language, cognitive, and emotional functions (Yoo and Kim, 2016; Thaut and Abiru, 2010; Van Vugt et al., 2016). Numerous studies have successfully integrated music therapy into stroke rehabilitation, demonstrating positive outcomes (Raglio et al., 2017a; Street et al., 2020).

Alongside this, research is actively being conducted in various academic fields to elucidate the mechanisms of music interventions and verify their effectiveness in motor rehabilitation (Devlin et al., 2019; Braun Janzen, et al., 2022; Sethi et al., 2017). While the growing evidence supporting various therapeutic techniques is a clear strength, issues such as the objectivity of terminology, consistency in intervention definitions, and insufficient description of stimulus characteristics have been repeatedly pointed out when the artistic medium of music is used as a research variable. In particular, small sample sizes, a lack of randomized controlled trials, and the absence of specific descriptions of music stimuli are reported as representative methodological limitations across music-based rehabilitation research (Devlin et al., 2019). Therefore, there is growing need for a more rigorous and clear methodological approach to apply music-based interventions to actual clinical settings (Grau-Sánchez et al., 2022).

Among the clinical populations for which music therapy is applied in neurorehabilitation, stroke patients occupy a particularly important position. Stroke is a leading cause of death and disability worldwide, and the number of patients continues to increase due to the aging population (GBD 2019 Stroke Collaborators, 2021). This has increased demand for rehabilitation services and has also generated interest in complementary approaches to functional recovery beyond conventional pharmacological and neuromodulation-based treatments (Juckett et al., 2020; Langhorne et al., 2011). In this context, music has been widely utilized in motor rehabilitation, and related research has rapidly increased as various technologies are developed based on neuroscience and music research findings.

However, the term “music-based intervention” used in stroke rehabilitation research encompasses a very broad range. Prior literature has also conceptualized music-based interventions broadly. Sihvonen et al. (2017) describe music-based interventions in neurological rehabilitation as including activities such as music listening, singing, and playing an instrument, while Magee et al. (2017) characterize music interventions in acquired brain injury rehabilitation as approaches used to stimulate brain functions involved in movement, cognition, speech, emotions, and sensory perception. More recent reporting guidance similarly defines music-based interventions broadly as the use of music or music-based experiences to address dimensions of health or human development. These broad formulations are useful for capturing the scope of the field, but they also highlight the need for greater conceptual precision when comparing studies that use qualitatively different forms of auditory input. Indeed, the literature identifies a wide range of approaches, from metronome-based auditory cues and simple rhythmic stimulation to instrumental training, improvisational interaction, and song-based communication training (Grau- Sánchez et al., 2013; Magee et al., 2017; Thaut et al., 2015; Zumbansen et al., 2014). Beyond music-centered approaches, sound-centered paradigms utilizing simple rhythmic tones, metronome pulses, and computer-based auditory feedback have also been widely reported. While these interventions all share the commonality of being auditory-based, their theoretical underpinnings, mechanisms of action, and clinical interpretation differ.

As Magee et al. (2017) and Grau-Sánchez et al. (2022) pointed out, the broad and complex nature of the concept of “music” can lead to conceptual ambiguity in research interpretation and hinder theoretical refinement. Specifically, if the components of “music” or “sound” defined in each study—acoustic characteristics, delivery method, and interaction context—are unclear, it becomes difficult to determine whether the studies are addressing the same intervention or exploring distinct therapeutic phenomena.

Geared by these concerns, this study aims to comprehensively map how music and sound have been defined, applied, and interpreted within the theoretical framework in stroke motor rehabilitation over the past 30 years. While previous scoping reviews have primarily focused on the effects of music-based interventions in stroke motor rehabilitation, we aim to define the conceptual scope of music-based interventions, analyze the characteristics of each intervention type, and propose scientific research directions to support the future therapeutic use of music in stroke motor rehabilitation.

## 2 Methods

This scoping review was conducted in accordance with the methodological guidance of the Joanna Briggs Institute (JBI) and reported following the Preferred Reporting Items for Systematic Reviews and Meta-Analyses extension for Scoping Reviews (PRISMA-ScR) (Peters et al., 2020). We aimed to map and critically examine how *music* and *sound* have been applied as therapeutic variables in post-stroke motor rehabilitation. Because prior literature has often used broad umbrella terms for music-based interventions, this review applied a more specific working operational distinction for the purpose of data charting and comparative analysis. In line with broader definitions in the literature, we regard music-based interventions as encompassing the use of music or music-based experiences in rehabilitation contexts. However, for this review, “music” was operationally defined as an organized auditory structure composed of multiple interrelated musical elements—such as rhythm, melody, harmony, tempo, and timbre—that together may convey perceptual, expressive, or affective meaning. “Sound” was operationally defined as non-musical or minimally structured auditory input, such as metronome pulses, single tones, or synthesized feedback, primarily used to cue, regulate, or provide feedback for movement. These definitions were used for analytic purposes to facilitate comparison across studies, rather than as absolute categories, and we acknowledge that some interventions may include overlapping features of both music and sound. This approach not only serves to define the scope of interventions, but also to understand how these conceptual and delivery differences impact the interpretation of therapeutic mechanisms.

### 2.1 Protocol and registration

This scoping review was conducted in accordance with the Joanna Briggs Institute methodology for scoping reviews and reported following the Preferred Reporting Items for Systematic Reviews and Meta-Analyses extension for Scoping Reviews. The review protocol was not prospectively registered. The eligibility criteria, screening procedures, data charting items, and synthesis approach were determined before data extraction and are described below. A completed PRISMA-ScR checklist is provided as a Supplementary File.

### 2.2 Search strategy and data sources

We conducted a comprehensive search of English-language articles in PubMed, CINAHL, Scopus, the Cochrane Library, PsycINFO, ProQuest, and Web of Science, with no restriction on publication date. The search strategy was structured around three conceptual domains: (1) neurological disorders, including stroke-related terms, including stroke and cerebrovascular accident; (2) auditory stimulation, including music, sound, rhythm, melody, listening, singing, and playing; and (3) rehabilitation and motor function, including rehabilitation, therapy, motor skills, upper extremity, and exercise. Keywords were combined using Boolean operators and adapted for each database. We also manually reviewed the reference lists of included studies to identify additional relevant records. The full database-specific search strategies, including Boolean operators and applied limits or filters, are provided in Supplementary Table S4.

### 2.3 Eligibility criteria

The inclusion criteria were developed using the Population–Concept–Context (PCC) framework recommended by JBI.

- Population: Individuals with stroke, regardless of type, severity, or phase of recovery.
- Concept: Use of *music* or *sound* as an independent or adjunctive variable in interventions targeting motor rehabilitation. Both upper- and lower-limb rehabilitation were included, encompassing interventions delivered by therapists, via devices, or through hybrid formats.
- Context: Any rehabilitation setting, including hospital-based, community-based, or home-based programs.

Studies were included if they presented empirical data—quantitative, qualitative, or mixed methods—examining the use of music or sound for motor rehabilitation after stroke. Review articles, editorials, dissertations, and commentaries were excluded.

### 2.4 Selection of sources of evidence

**Figure 1:**
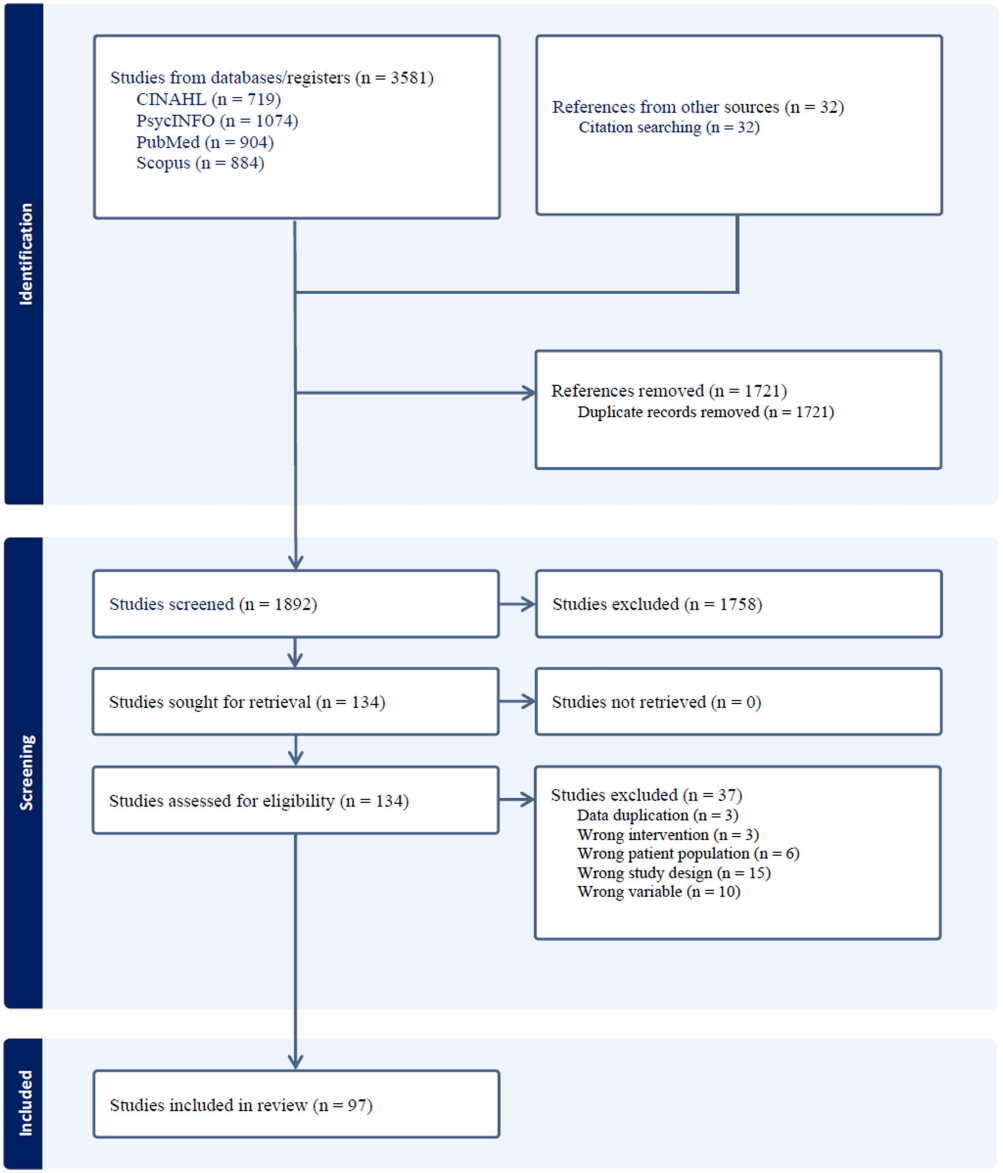
PRISMA flow diagram illustrating the study selection process for the scoping review. Of 3,613 records identified, 1,721 duplicates were removed. Following title and abstract screening and full-text assessment, 97 studies were included in the final review.

All retrieved citations were imported into Covidence for citation management, duplicate removal, and screening. After duplicates were removed, two independent reviewers screened titles and abstracts against the eligibility criteria. Potentially relevant studies were then assessed through full-text review by two independent reviewers. Disagreements at each stage were resolved through discussion or consultation with another reviewer. Reasons for exclusion at the full-text stage were recorded and are reported in the PRISMA flow diagram (Figure 1).

### 2.5 Data charting process

Data were charted using a standardized Excel-based data charting form developed specifically for this scoping review. The form captured bibliographic information, study design, data collection approach, participant characteristics, stroke-related characteristics, setting, intervention or non-intervention characteristics, provider involvement, outcome measures, measurement timepoints, key findings, and detailed information on the use of music or sound, including selection, musical or auditory features, delivery method, and implementation-related challenges.

Data charting was conducted by independent reviewers. To enhance consistency, the review team discussed the charting items and resolved discrepancies through discussion or consultation with another reviewer when necessary. The standardized data charting form is provided as Supplementary Table S5.

### 2.6 Critical appraisal of individual sources of evidence

Formal critical appraisal of the included studies was not conducted. This decision was made because the objective of this scoping review was to map the range, characteristics, terminology, and methodological variability of music- and sound-based approaches in post-stroke motor rehabilitation, rather than to evaluate intervention effectiveness or exclude studies based on methodological quality. Accordingly, the findings are presented as a descriptive and thematic synthesis of the available evidence, without weighting the results according to methodological quality.

### 2.7 Synthesis of results

The extracted data were synthesized using both descriptive numerical summary and thematic synthesis. First, the general characteristics of the included studies were summarized according to publication year, country, study design, participant characteristics, stroke stage, and targeted motor functions. Studies were then categorized according to whether they involved interventional or non-interventional designs, and whether music, sound, or both were used as the primary auditory component.

For studies involving music- or sound-based approaches, we further summarized intervention or task characteristics, including the name and content of the intervention or task, session length, delivery format, setting, provider discipline, and the way music or sound was selected, structured, and delivered. Particular attention was given to how musical or auditory parameters were reported, including rhythm, melody, tempo, metronome cues, live or recorded delivery, computer-generated sound, device-based feedback, and participant- or researcher-selected materials.

Given the heterogeneity of study designs, interventions, auditory stimuli, and outcome measures, no quantitative synthesis or meta-analysis was conducted. Instead, the findings were collated narratively to identify patterns, variations, and reporting gaps in the use of music and sound as research variables in post-stroke motor rehabilitation. Thematic synthesis was conducted to identify recurring methodological issues, including terminology use, specification of musical or auditory components, intervention delivery, provider involvement, and implications for clinical replicability and future research.

## 3 Results

A total of 97 studies were included from 1993 to 2023, indicating a consistent rise in international interest in the use of auditory stimulation in stroke motor rehabilitation. The number of studies has increased significantly since 2010, reflecting an expanding body of evidence on auditory-motor entrainment and neuroplasticity (Thaut and Abiru, 2010; Särkämö and Soto, 2012). While the included studies illustrate diverse conceptualizations and applications of music and sound, they also reveal significant differences in definitions, delivery methods, and theoretical rationale.

### 3.1 Overview of study characteristics

The included studies originated from 19 countries, with the United States (*n* = 24) and South Korea (*n* = 19) contributing the largest numbers, followed by Germany, the United Kingdom, Spain, and Canada (Supplementary Table S1; see asterisked references). Most were quantitative in design and employed interventional clinical trials (*n* = 71), while non-interventional or observational approaches (*n* = 26) were less common (Supplementary Tables S2, S3). Over time, the composition of research teams shifted from being primarily led by physical or occupational therapists to including more interdisciplinary collaborations involving neuroscientists, engineers, and music therapists. This trajectory indicates an expanding methodological and disciplinary base for music-related rehabilitation research.

### 3.2 Conceptual diversity in the use of music

Across the 97 studies included, 56 used musically structured material, 47 employed non-musical sound, and six incorporated both forms within a single protocol, as summarized in Table 1. The auditory material categorized as “music” ranged from simple isochronous beats or metronome-like pulses to harmonically rich or melodic structures. In contrast, “sound” typically referred to rhythmic cueing, single tones, or tonal feedback without musical organization. The presence of studies using both modalities illustrates that the distinction between music and sound is not fixed but shifts across research contexts.

**Table 1:** Comparative analysis of auditory modality, delivery type, and professional involvement.

Auditory modality	Delivery method	Provider involvement	Type of clinical trials	Included studies
Music (*n* = 56)	Human-delivered (*n* = 40)	MT (*n* = 7)	ICT (*n* = 7)	Chong et al. (2017), Haire et al. (2021), Raglio et al. (2017b), Ruotsalainen et al. (2022), Shin et al. (2015), Street et al. (2018), and Street et al. (2019)
		MT + others (*n* = 7)	ICT (*n* = 7)	Donoso Brown et al. (2021), Fujioka et al. (2018), Gonzalez-Hoelling et al. (2021), Jun et al. (2013), Mainka et al. (2018), Raghavan et al. (2016), and Silveira et al. (2018)
		Non-MT (PT, OT etc.) (*n* = 5)	ICT (*n* = 5)	Bunketorp-Käll et al. (2017), Grau-Sánchez et al. (2018), Thaut et al. (1997), Villeneuve et al. (2014), and Young et al. (2021)
		Not specified (*n* = 21)	ICT (*n* = 16)	Altenmüller et al. (2009), Amengual et al. (2013), Bunketorp-Käll et al. (2019), Fotakopoulos and Kotlia (2018), Ghai et al. (2021), Grau- Sánchez et al. (2013), Grau-Sánchez et al. (2017), Ripollés et al. (2016), Schneider et al. (2007), Schneider et al. (2010), *Thaut et al. (2007), Tong et al. (2015), Van Vugt et al. (2014), Villeneuve and Lamontagne (2013), *Wang et al. (2021), and Wright et al. (2017)
			NCT (n = 5)	*Crosby et al. (2020), *Kang et al. (2020), Lee S. Y. et al. (2018), *Peyre et al. (2020), and Thaut et al. (1993)
	Device (*n* = 16)	Device only (*n* = 5)	ICT (*n* = 4)	Adamovich et al. (2009), Nikmaram et al. (2019), Scholz et al. (2016), and Zondervan et al. (2016)
			NCT (*n* = 1)	Collimore et al. (2023)
		Device + provider (*n* = 11)	ICT (*n* = 8)	Friedman et al. (2014), Hankinson et al. (2022), Hutchinson et al. (2020), Kirk et al. (2016), Raglio et al. (2021), Sanders et al. (2020), Schauer and Mauritz (2003), and Segura et al. (2021)
			NCT (*n* = 3)	*Aluru et al. (2014), Douglass-Kirk et al. (2023), and Kantan (2022)
Sound (*n* = 47)	Human-delivered (*n* = 40)	Non-MT (PT, OT etc.) (*n* = 17)	ICT (*n* = 11)	Chouhan et al. (2012), Kim et al. (2012), Lee S. et al. (2018), Malcolm et al. (2009), McCue et al. (2022), Luft et al. (2004), Park et al. (2015), Park and Chung (2018), Shahine and Shafshak (2014), Song (2015), and Suh et al. (2014)
			NCT (*n* = 6)	Kantan et al. (2023), Kim et al. (2014), Ko et al. (2016), Mizuta et al. (2022), Lee et al. (2012), and Prassas et al. (1997)
		Not specified (*n* = 23)	ICT (*n* = 12)	Choi and Kim (2021), Kim et al. (2011), Kim and Oh (2012), Mishr and Jose (2022), Richards et al. (2008), Shaw et al. (2022), *Thaut et al. (2007), Tian et al. (2020), Van Delden et al. (2013), *Wang et al. (2021), Whitall et al. (2000), and Whitall et al. (2011)
			NCT (*n* = 11)	Cha et al. (2014), *Crosby et al. (2020), Ford et al. (2007), Jung et al. (2012), *Kang et al. (2020), Pelton et al. (2010), *Peyre (2020), Roerdink et al. (2007), Sethi et al. (2017), Thaut et al. (2002), and Wright et al. (2016)
	Device (*n* = 7)	Device only (*n* = 3)	NCT (*n* = 3)	Chen et al. (2016), Roerdink et al. (2009), and Secoli et al. (2011)
		Device + provider (*n* = 4)	ICT (*n* = 3)	Beckelhimer et al. (2011), Hsu et al. (2012), and Muto et al. (2012)
			NCT (*n* = 1)	*Aluru et al. (2014)

Studies marked with an asterisk incorporated both music and sound. ICT = interventional clinical trial; MT = music therapist; NCT = non-interventional clinical trial; OT = occupational therapist; PT = physical therapist.

Despite this variability, few studies provided detailed descriptions of musical or acoustic parameters such as tempo, meter, timbre, or harmonic complexity. As a result, interventions labeled under the same auditory category often remain difficult to compare. This limited specification continues to generate conceptual ambiguity in the literature and complicates attempts to interpret how auditory stimuli function within stroke motor rehabilitation.

### 3.3 Delivery mechanisms and provider involvement

Delivery methods differed notably across disciplines. As summarized in Table 1, studies employing musically structured material were predominantly interventional clinical trials, with 47 of 56 music studies (84%) classified as ICTs and only nine (16%) categorized as NCTs. Sound-based studies showed a similar, through more moderate pattern, with 31 of 47 studies (66%) identified as ICTs and 16 (34%) as NCTs. Across both auditory modalities, ICT studies commonly adopted hybrid delivery models in which therapists provided direct guidance or combined their input with device-based systems, whereas NCT designs relied more heavily on equipment-based or researcher-operated protocols without real-time therapeutic adjustment.

Studies that included music therapists (approximately 15% of all music-involved studies) tended to structure sessions in a real-time, adaptive manner, drawing on improvisation, emotional attunement, and moment-to-moment interaction with patients. By contrast, studies led by physical therapists, engineers, or researchers focused more narrowly on enhancing motor accuracy through pre-recorded rhythmic cues or algorithm-generated auditory feedback. These distinctions reflect not only procedural differences but also the broader disciplinary assumptions that shape how auditory material—whether music or sound—is conceptualized and operationalized within therapeutic interventions.

### 3.4 Functional and emotional outcomes

Upper-limb rehabilitation was the most frequent target (55 studies), followed by lower-limb training focusing on gait and balance (47 studies). Five studies included interventions involving both upper-limb and lower-limb rehabilitation (Supplementary Table S1). Interventions commonly included piano or drum-pad exercises, rhythmic stepping, or auditory cueing for gait modulation. While most studies assessed motor outcomes such as movement speed, coordination, or balance, 12 studies (12%) incorporated psychosocial variables including motivation, mood, or self-reported recovery. In these studies, improvements in both motor and emotional domains were often attributed to the motivational and affective engagement inherent in musical participation.

### 3.5 Empirical basis for a conceptual continuum

**Figure 2:**
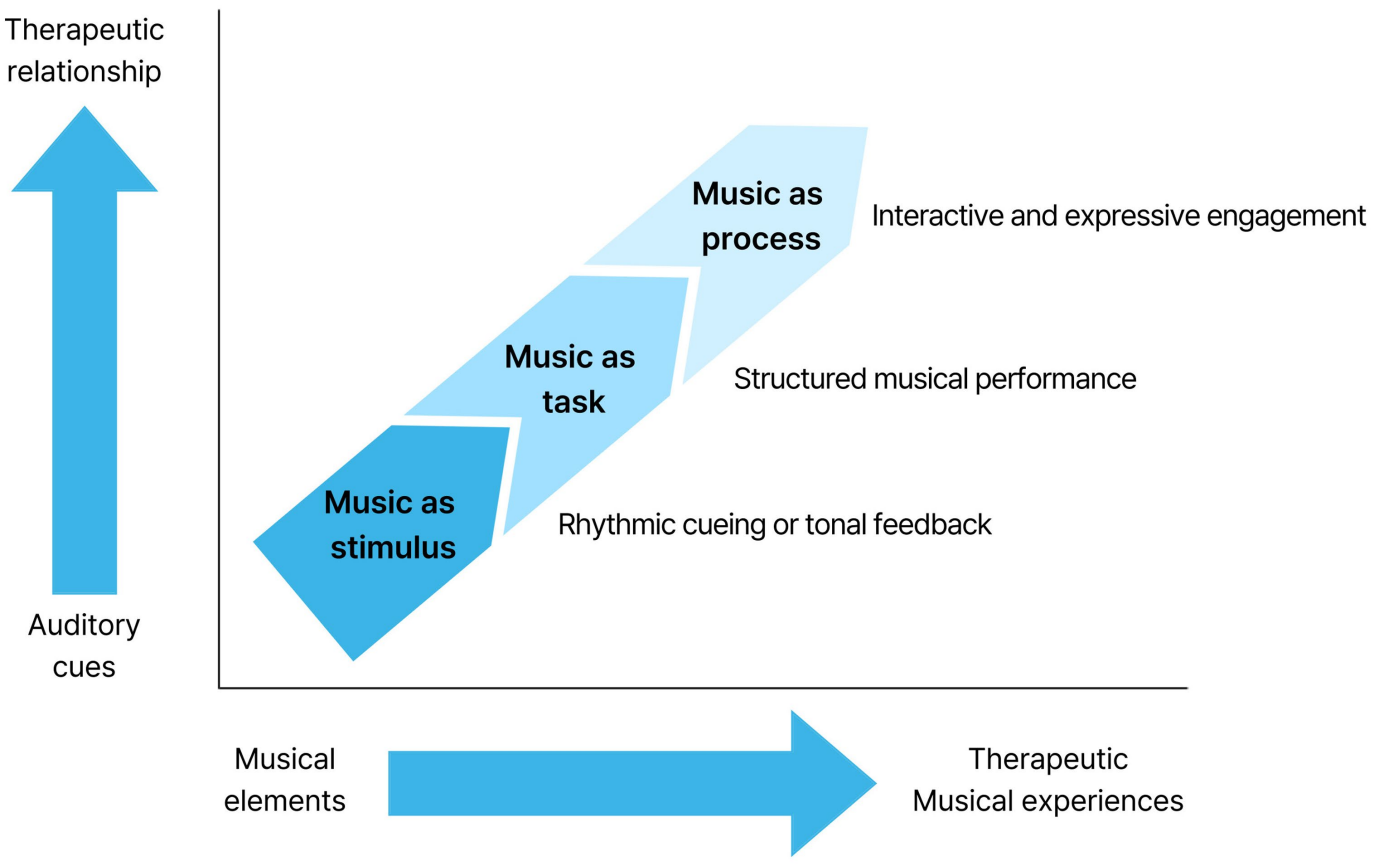
Conceptual continuum of music-based approaches in stroke motor rehabilitation, ranging from Music as stimulus (rhythmic cueing or tonal feedback) to Music as task (structured musical performance) to Music as process (interactive and expressive engagement), reflecting increasing levels of musical complexity and therapeutic relational engagement.

Based on a comparative analysis of auditory modality, delivery method, and expert involvement (Table 1), interventions could be broadly categorized into three categories: music stimulation, music tasks, and music processes (Figure 2).

Music stimulation uses short rhythmic or tonal cues to guide the timing and pacing of movement. Most sound-based or device-based studies fall into this category, relying on sensorimotor entrainment as the primary mechanism. In this approach, music functions mainly as repetitive auditory feedback that helps stabilize movement patterns.

The music task approach structures musical experiences into a single, performable activity. Structured musical activities, such as playing an instrument or moving to a melody, serve as a framework for the motor movements necessary for physical rehabilitation. This process enhances motor training effectiveness by adding attention, sensory stimulation, and motivation to the repetitive musical behavior. The analysis of this study revealed that many studies provided a hybrid approach, using either verbal instructions from the therapist or music as feedback.

The music process-based approach treats music experiences as part of a therapeutic interaction. Emotional attunement, relationship building, and musical exchange between therapist and patient are central elements, most often provided by the music therapist. In this approach, music is not simply an auditory cue, but rather functions as a process that fosters communication, regulation, and participation.

These three approaches are not separate categories, but rather lie on a continuum, extending from external stimulus-centered training to interaction-centered treatment (Figure 2). In this study, approximately two-thirds of the studies focused on stimulus-task-based approaches utilizing rhythmic entrainment and repetitive training, while relatively few studies focused on emotional relationships and process approaches that examine the holistic effects of music. This suggests the need for an integration of training models based on neurophysiological responses with models that emphasize psychosocial experiences.

## 4 Discussion

This scoping review examined how music and sound have been used in stroke motor rehabilitation research over the past 30 years. Although the number of studies has grown steadily, the field has not advanced with the same level of conceptual clarity. The term music-based intervention is still used as a single category, even though it encompasses approaches as different as rhythmic cueing, structured music tasks, and therapist-led music therapy. These approaches serve different purposes and operate through different mechanisms, suggesting that the continued use of one broad term requires careful re-evaluation. This inconsistency is not simply a matter of wording; it points to a deeper divide between viewing music as a physiological stimulus and viewing it as a therapeutic, relational process.

A problem identified throughout the research is the lack of clarity in the definition of music. As several scholars have pointed out (Magee et al., 2017; Grau-Sánchez et al., 2022), the term “music” is widely used in rehabilitation research without a specific description of the structure or theoretical basis of musical stimulation. In this study, music- or sound-based interventions ranged from simple beat cues to complex musical materials, but few studies described them in a comparable manner. This ambiguity leads to the problem of different interventions being assumed to work in the same way, and when the components, delivery method, and context of the stimulus are not clearly reported, interpretation of results and integration across studies become difficult. To address these issues, Robb and colleagues developed a reporting checklist and accompanying guide (Robb et al., 2025), emphasizing the importance of clearly outlining key details of music-based interventions.

The three categories of “stimulus-task-process” presented in this study help understand these differences from both functional and theoretical perspectives. Although originally conceptualized across various motor and neurological conditions, these categories are useful for interpreting how music-based interventions function specifically within stroke motor rehabilitation. First, *music as a stimulus* is an approach that uses rhythmic auditory cues as external modulators to align movement with timing and synchronization (Thaut and Abiru, 2010; Chen et al., 2008). While it has been repeatedly demonstrated to improve gait and coordination, it is characterized by its reliance on external cues rather than internal engagement. Second, *music as a task* utilizes the musical activity itself as a framework for motor practice, similar to instrument-based repetitive training (Altenmüller et al., 2009; Amengual et al., 2013). This approach leverages the structure and sensory elements provided by music to simultaneously enhance motor learning and motivation. Third, *music as a process* centers on therapist-patient interaction and emotional communication (Clements-Cortes and Bartel, 2018; Geretsegger et al., 2014). Music is not simply a stimulus; it becomes an experiential and relational context that encompasses emotion regulation, relationship building, and co-creation.

These three are not separate categories, but rather a continuum from approaches closer to external regulation to interactive and adaptive approaches. Most literature focuses on the stimulus or task level, both of which are easily controllable and measurable. Despite the growing emphasis on emotional and social engagement (Fancourt and Finn, 2019; Altenmüller and Furuya, 2016), process-centered approaches have received relatively little attention in stroke rehabilitation research. This reflects a larger interdisciplinary gap between the causal precision favored by neuroscience and the meaningfulness of subjective experience that is crucial for therapeutic practice. Therefore, an integrative framework that considers both the structural and measurable elements of sound and the experiential and affective aspects of music is needed to fully capture how music-based interventions operate within stroke motor rehabilitation.

A broader pattern emerging from the mapped literature is that sound-based studies were more often associated with externally paced gait or movement training, whereas musically structured interventions more frequently incorporated instrumental practice, motivational support, or therapist-guided adaptation. In addition, psychosocial outcomes were assessed in only a small subset of studies, suggesting that the field has largely prioritized measurable motor performance over broader therapeutic experience. This imbalance does not diminish the relevance of motor outcomes, but it does indicate that the experiential, motivational, and relational dimensions of music remain comparatively underrepresented in stroke motor rehabilitation research.

Emerging hybrid approaches demonstrate the potential for integrating technology-based stimulation with therapeutic interaction. For example, studies combining algorithmically generated auditory feedback with real-time therapist intervention (Raglio et al., 2021; Friedman et al., 2014) demonstrate the complementary nature of the precision provided by the machine and the relational attunement provided by the therapist. This approach bridges the gap between machine- and relationship-centered approaches, maintaining structural control over the intervention while enabling interactions that flexibly adapt to patient responses. Although these examples come from broader clinical contexts, they highlight principles that are highly relevant to stroke motor rehabilitation, where both precise motor entrainment and individualized therapeutic engagement are essential as supported by our findings. This is consistent with the view that music is a neuroemotional system in which rhythm and affective processes operate in concert (Mohammad Alipour et al., 2019; Trost and Vuilleumier, 2013).

The identified continuum from sound-based cueing to relational music engagement has important clinical implications. Rather than representing discrete categories, these approaches may be understood as hierarchically related, with rhythmic auditory cueing serving as a foundational mechanism for motor timing and coordination, upon which more complex, interactive, and relational forms of music engagement may be built. Such progression may support not only motor performance, but also motivation, engagement, and functional integration into daily life. From a clinical perspective, this framework may inform more precision-oriented rehabilitation approaches, in which intervention strategies are selected, tailored, and potentially layered according to individual patient needs, therapeutic goals, and rehabilitation context.

Across the included studies, methodological heterogeneity was evident not only in the auditory materials used, but also in sample size, study design, control conditions, intervention dosage, outcome domains, and the level of detail reported for musical parameters. Although formal quality appraisal was beyond the scope of this review, these patterns are important because they limit direct comparison across studies and complicate interpretation of the relative contribution of sound-based cueing, music-involved task practice, and therapist-led music processes. More rigorously designed trials with clearer intervention reporting and more comparable outcome selection are needed to strengthen the interpretability of this literature.

Future research should directly engage with several issues raised in this review. First, musical and acoustic parameters—including tempo, rhythmic structure, and dynamics—must be reported with greater precision to support meaningful comparison and replication. Second, studies need to provide clear explanations of why specific auditory stimuli were chosen, how they were applied, and how the findings should be interpreted. Finally, therapist-led, researcher-led, and device-centered approaches rest on different assumptions and mechanisms, which can shape clinical outcomes in distinct ways. For this reason, the expertise and roles of those delivering the intervention should be described with far greater clarity.

Distinguishing interventions along the stimulus-task-process continuum will facilitate a clearer understanding of the mechanisms involved in entrainment, learning, and relational engagement in future reviews and meta-analyses. This study emphasizes that while music and sound possess distinct properties, they are interconnected and function as therapeutic elements in stroke motor rehabilitation. Thus, the purpose of this framework from our findings is not intended to categorize interventions, but rather to reduce conceptual confusion and facilitate a more accurate interpretation of how auditory-based stimuli contributes to stroke motor rehabilitation processes.

This review has several limitations. First, only English-language publications were included, which may have excluded relevant studies published in other languages. Second, although a broad range of databases was searched, unpublished reports and other forms of grey literature were not included. Third, formal critical appraisal was not conducted because the purpose of this scoping review was to map the scope and methodological variability of the literature rather than to assess intervention effectiveness. Finally, the classification of music and sound was based on the information reported in the included studies; therefore, studies with insufficient descriptions of auditory stimuli may have been difficult to categorize precisely.

## 5 Conclusion

The artistic qualities of music must be defined clearly and objectively when used in therapeutic contexts. As music-based intervention continues to develop as an independent domain within stroke neurorehabilitation, it is essential to specify the musical elements, mechanisms, and modes of application through precise and detailed research descriptions. Greater methodological transparency is also needed to clarify the boundaries between music and sound and to reduce remaining conceptual ambiguities. These developments will strengthen the scientific foundations of music therapy and support a more consistent evidence base for the therapeutic use of music in stroke rehabilitation.

## Supplementary material

SUPPLEMENTARY Table S1 General characteristics of included studies (*n* = 97), including country, study design, participant characteristics, targeted motor functions, and stroke stage.

SUPPLEMENTARY Table S2 Music and sound variable analysis for interventional studies (*n* = 71), including intervention content, session details, provider involvement, and auditory stimulus characteristics.

SUPPLEMENTARY Table S3 Music and sound variable analysis for non-interventional studies (*n* = 26), including task content, session details, provider involvement, and auditory stimulus characteristics.

SUPPLEMENTARY Table S4 Full database-specific search strategies, including Boolean operators, search strings, and applied filters for all seven databases.

SUPPLEMENTARY Table S5 Standardized data charting form used for data extraction across included studies. We note that this file was not reflected in the system and is hereby re-uploaded to the Production Forum. Please ensure that this file is published alongside the other supplementary materials.

SUPPLEMENTARY File 6 Completed PRISMA-ScR checklist.
