# Supplementary material for: Reconsidering music in stroke rehabilitation: a scoping review from auditory stimulus to relational process
Source: Front Psychol. 2026 Jun 5;17:1774971. doi: 10.3389/fpsyg.2026.1774971 (PMC13279217; doi:10.3389/fpsyg.2026.1774971)
Supplement: SUPPLEMENTARY Table S1 — General characteristics of included studies (n = 97), including country, study design, participant characteristics, targeted motor functions, and stroke stage. [file Data_sheet_1.pdf]

Support Information Table S1. General characteristics of studies

First author (Year)	Country	Study design	Participants		Targeted function(s)	
			N (Age: mean or range, Gender)	Stage of stroke	Primary (motor)	Secondary (non-motor)
Thaut (1993)	USA	Cross-sectional study	10 (70±10.4, 8M:2F)	Subacute, chronic	LL (gait)	–
Prassas (1997)	USA	Within-subject study (repeated measures)	I: 8 (49-87, 7M/1F)	Not indicated	LL (gait, functional movement)	–
Thaut (1997)	USA	RCT	I: 10 (73±7, 13M/8F)<br>C:10(72±8, 5M/5F)	Acute	LL (gait)	–
Whitall (2000)	USA	One-group pre/post study	I: 16 (44-89, 8M/8F)	Chronic	UL (arm)	Psychosocial (QoL, fatigue)
Thaut (2002)	USA	Within-subject study (cross-over design)	21 (52.7±13.7, 13M/8F)	Subacute, chronic	UL (arm)	–
Schauer (2003)	Germany	RCT	I:11 (59±11, Not indicated)<br>C:12 (61±12, Not indicated)	Subacute	LL (gait)	–
Luft (2004)	USA	RCT	I: 9 (63.3±15.3, 7M/2F)<br>C: 12 (59.6±10.5, 5M/7F)	Chronic	UL (arm)	–
Ford (2007)	USA	Within-subject study (repeated measures)	I: 11 (14-78, 10M/1F)	Not indicated	LL (gait)	–
Roerdink (2007)	Netherlands	Non-RCT	I: 11 (60, 7M/4F)<br>C(healthy group): 10 (60, 6M/4F)	Not indicated	LL (gait)	–
Schneider (2007)	Germany	Non-RCT	I: 20 (58.1±9.9, 12M/8F)<br>C(healthy group): 20 (4.5±10.2, 15M/5F)	Subacute	UL (hand, arm)	–
Thaut (2007)	USA	RCT	I: 43 (69.2±11, 22M/21F)<br>C: 35 (69.7 ± 11, 19M/16F)	Not indicated	LL (gait)	–
Richard (2008)	USA	One-group pre/post study	I: 15 (64.4, 6M/9F)	Chronic	UL (hand, arm)	–
Adamovich (2009)	USA	One-group pre/post study (proof of concept)	I: 4 (68-78, 2M/2F)	Chronic	UL (finger)	–
Altenmüller (2009)	Germany	Non-RCT	I: 32 (55.7 ±12.3, 16M/16F)<br>C: 30 (53±11.8, 24M/6F)	Chronic	UL (finger, hand, arm)	Cognition (executive function)
Malcolm (2009)	USA	One-group pre/post study	I: 5 (72.8±6.5, 5M)	Chronic	UL (arm, shoulder)	–
Roerdink (2009)	Netherlands	One-group pre/post study	I: 9 (58.3±12.3, 6M/3F)	Chronic	LL (gait)	–
Pelton (2010)	UK	Within-subject study (repeated measures)	I: 8 (70, 5M/3F)	Not indicated	LL (gait)	–
Schneider (2010)	Germany	Non-RCT	I: 32 (55.7 ± 12.3, 16M/16F)<br>C1(constraint-induced therapy): 15 (56.1 ± 10.7, 10M/5F)<br>C2(conventional treatment only): 30 (53 ± 11.8, 24M/6F)	Subacute	UL (arm)	–

Beckelhimer (2011)	USA	Case study	I: 2 (P1: 68, M, P2:75, M)	Not indicated	UL (arm)	–
Kim (2011)	South Korea	RCT	I: 15 (60.07±11.93, 7M/8F)	Chronic	LL (gait)	–
Secoli (2011)	USA	Non-RCT	I: 33 (left-side hemiparesis: 14 (56.3±12.3, 6M/8F)/right-side hemiparesis: 5 (61.8 ± 5.0, 4M/1F)<br>C(healthy group): 14 (27 ± 7.53, 11M/3F)	Not indicated	UL (arm)	–
Whitall (2011)	USA	RCT	I: 42 (59.8±9.9, 26M/16F)<br>C (DMTE): 50 (57.7±12.5, 24M/26F)	Chronic	UL (arm)	–
Chouhan (2012)	Australia	RCT	I: 15 (56.73±5.99, 12M/3F)<br>C1 (visual cueing): 15 (58.13±4.14, 12M/3F)<br>C2 :15 (57.33±5.51, 12M/3F)	Acute	UL(hand), LL (gait, balance)	–
Hsu (2012)	Taiwan	Non-RCT	I: 14 (57.86 ± 12.03, 9M/5F)<br>C(healthy group): 14(age-marched, 9M/5F)	Not indicated	UL(hand)	–
Jun (2013)	South Korea	One-group pre/post study	I: 15 (60.7±8.59, 6M/9F)	Not indicated	UL (shoulder, elbow)	Psychosocial (mood)
Jung (2012)	South Korea	One-group pre/post study	I: 12 (52.58±12.42, 7M/5F)	Chronic	LL (gait)	–
Kim, J. H. (2012)	South Korea	RCT	I: 10 (58.3±11.8, 6M/4F)<br>C: 10 (51.8±13.7, 7M/3F)	Subacute	LL (gait, balance, functional movement)	–
Kim, J. S. (2012)	South Korea	RCT	I: 10 (65.2±6.8, Not indicated)<br>C: 10 (64.5±8.1, Not indicated)	Chronic	LL (gait)	–
Lee (2012)	South Korea	One-group pre/post study	I: 25 (64.31±8.2, 14M/11F)	Chronic	LL (gait)	–
Muto (2012)	Germany	Non-RCT	I: 8 (56.75±16.14, 5M/3F)<br>C: 8 (57.88±11.93, 5M/3F)	Acue, Subacute, Chronic	LL (gait)	–
Amengual (2013)	Spain	Non-RCT	I: 19 (59.5, 14M/5F)<br>C(healthy group): 14 (56.9, 12M:2F)	Chronic	UL	–
Grau-Sánchez (2013)	Spain	Non-RCT	I: 9 (61.8, 6M/3F)<br>C(healthy group): 9 (59.3, 7M/2F)	Not indicated	UL (hand, arm)	Psychosocial (QoL)
Van Delden (2013)	Netherlan ds	RCT	I: 19 (62.6±9.8, 11M/8F)<br>C1(mCIMT): 22 (59.8±13.8, 14M/8F)<br>C2 (DMCT): 19 (56.9±12.7, 16M/3F)	Not indicated	UL (hand, arm)	–
Villeneuve (2013)	Canada	One-group pre/post study	I: 3 (58~67, 3M)	Chronic	UL (finger, hand)	–
Aluru (2014)	USA	One-group pre/post study	I: 20 SP: 7 (51.6±11.2, 4M/3F)<br>SC: 6 (55.3±19.3,3M/3F)<br>MP: 7 (55.9±12.5, 5M/2F)	Chronic	UL (hand)	–

Cha (2014)	South Korea	Cross-sectional study	I: 41 (60.8±19.8, 24M/17F)	Not indicated	LL (gait)	–
Friedman (2014)	USA	One-group pre/post study	I: 12 (57±30.5, 7M/5F)	Chronic	UL (hand)	–
Kim (2014)	South Korea	One-group pre/post study	I: 16 (49.2, 9M/7F)	Chronic	UL (elbow)	–
Shaine (2014)	Egypt	RCT	I: 36 (60.73, 39M/37F)<br>C:36 (62.7, 19M/17F)	Chronic	UL	–
Suh (2014)	South Korea	RCT	I: 24 (57.8±11.6, Not indicated)<br>C: 24(57.2±12.5 Not indicated)	Chronic	LL (gait, balance)	–
Van Vugt (2014)	Germany	RCT	I: 14 (65.6±10.5, 6F/8M)<br>C: 14 (67.1±11.8, 10F/4M)	Not indicated	UL(hand)	Psychosocial (mood)
Villeneuve (2014)	Canada	One-group pre/post study	1: 13 (41-79, 8M/5F)	Not indicated	UL(finger, hand, arm)	–
Park (2015)	South Korea	RCT	I1(TRAS): 19 (51.8, 4M/5F)<br>I2(ORAS): 55, 6M/4F)	Chronic	LL (gait)	–
Shin (2015)	South Korea	One-group pre/post study	I: 11 (44.27±7.04, 7M/4F)	Chronic	LL (gait)	–
Son (2015)	South Korea	Non-RCT	I: 20 (48.65±12.81, 13M/ 7F)<br>C: 20 (51.15±14.81, 11M/9F)	Not indicated	UL (hand)	–
Tong (2015)	China	RCT	I: 15 (50.1 ±14.8, 13M/2F)<br>C: 15 (48.6 + 14.6; 13M/2F)	Not indicated	UL (hand, arm)	–
Chen (2016)	USA	Case series	I: 5 (57.6, 4M/1F)	Not indicated	UL	–
Kirk (2016)	UK	Case study (feasibility)	I: 3 (P1: 50, M, P2: 44, F, P3: 50, M)	Chronic	UL	–
Ko (2016)	South Korea	Within-subject study (repeated measures)	I: 15 (56.0±7.4, 11M/4F)	Chronic	LL (gait)	–
Raghavan (2016)	USA	One-group pre/post study	I: 13 (52±14, 9M/4F)	Chronic	UL (hand)	–
Ripollés (2016)	Spain	Non-RCT	I: 20(59.1±9.04, 17M:3F)<br>C(healthy group): 14(56 ± 9.6, 12M/2F)	Chronic	UL (hand, arm)	–
Scholz (2016)	Germany	RCT	I: 15 (32-86, 8M/7F)<br>C: 10 (57-85, 6M/4F)	Not indicated	UL (arm)	–
Wright (2016)	UK	Case study	1 (81, F)	Not indicated	LL (gait)	–
Zondervan (2016)	USA	RCT	I: 9 (60 (45–74), 5M/4F)<br>C: 8 (59 (35–74), 5M/3F)	Not indicated	UL (hand)	–
Bunketorp-Kall (2017)	Sweden	RCT	I: 41 (62.7± 6.7, 23M/18F)<br>C1(horse riding): 41 (62.6± 6.5, 24M/17F)<br>C2: 41 (63.7± 6.7, 22M/19F)	Chronic	UL (arm)	Psychosocial (QoL, perception of recovery)
Chong (2017)	South Korea	One-group pre/post study	I: 27(55.13M/3F)	Not indicated	LL (gait, balance, functional movement)	Psychosocial (QoL)
Grau-S´anchez (2017)	Spain	RCT	1:19 (60.1, 11M/8F)<br>C:20 (62.5, 12M/8F)	Subacute	UL (hand, arm)	Cognitive, psychosocial (mood, QoL)
Raglio (2017)	Italy	RCT	I: 19 (70.4±8.9, 8M/11F)<br>C: 19 (75.4±=7.6, 8M/11F)	Acute	UL (finger, hand)	Psychosocial (anxiety, depression)

Sethi (2017)	USA	RCT	I: 10 (67 ± 8.9, 9M/1F)<br>C(healthy group): 9 (57 ± 6.49, 1M/8F)	Chronic	UL (finger, hand, shoulder, elbow)	–
Wright (2017)	UK	Within-subject study (cross-over design)	I: 7<br>C: 5<br>(Total-56±11, 9M/4F)	Chronic	LL (gait, functional movement)	–
Fotakopoulos (2018)	Greece	RCT	I: 24 (73.29 ± 4, 14M/10F)<br>C: 41 (76.02 ± 3, 19M/22F)	Acute	LL (functional movement)	Cognitive
Fujioka (2018)	USA	RCT	I: 14 (64.2±9.4, 9M/5F)<br>C: 14 (54.3±11.3, 11M/3F)	Chronic	UL (hand, arm)	Cognitive, psychosocial (mood, QoL)
Grau-S´anchez (2018)	Spain	Case study	1 (55, M)	Chronic	UL (hand, arm)	–
Lee, S. (2018)	South Korea	RCT	I: 23 (56.00±9.39, 13M/10F)<br>C: 21 (54.92±6.65, 11M/10F)	Chronic	LL (gait, balance)	–
Lee, S. Y. (2018)	South Korea	One-group pre/post study	I: 20 (55.6±14.1, 8M/12F)	Chronic	LL (gait)	–
Mainka (2018)	Germany	RCT	I: 11 (63.7±8.8, 7M/4F)<br>C1 (treadmill training): 13 (65.6±8.5, 11M/2F)<br>C2 (NDT): 11 (61.1±8.6, 8M/3F)	Subacute	LL (gait, balance)	–
Park (2018)	South Korea	Non-RCT	I: 12 (55±5, 3M/2F)<br>C1(visual feedback): 12 (52.4±12.05, 3M/2F)<br>C2(PT only): 16 (57.2±11.56, 2M/3F)	Not indicated	LL (gait)	–
Silveira (2018)	Australia	Case study	1 (74, F)	Acute	UL (finger)	–
Street (2018)	UK	RCT	I: 6 (53.2, 2M/4F)<br>C: 5 (67.6, 3M/2F)	Subacute, chronic	UL	–
Bunketorp-Kall (2019)	Sweden	RCT	I: 41 (62.7±6.7, 23M/18F)<br>C1(horse-riding): 41 (62.6±6.5, 24M/17F)<br>C2: 41 (63.7±6.7, 22M/19F)	Subacute	LL (gait, functional movement)	–
Nikmaram (2019)	Germany	RCT	I: 7 (65.30±12.70, 6M/1F) /14 (68.71±11.76, 10M/4F)<br>C: 5 (66.40±6.90, 3M/2F) / 14 (70.21±14.29, 11M/3F)	Acue, Subacute, Chronic	UL (arm)	–
Street (2019)	UK	Case study	C1: 1 (74, F)<br>C2: 1 (61, M)	C1: subacute; C2: chronic	UL (finger, hand, elbow)	–
Crosby (2020)	Canada	One-group pre/post study	I: 22 (61.5±10.4, 15M/7F)	Not indicated	LL (gait)	–
Hutchinson (2020)	USA	One-group pre/post study (proof-of-concept study)	I: 11 (57.7, 9M/2F)	Chronic	LL (gait)	–
Kang (2020)	South Korea	One-group pre/post study	I: 18 (49.78±15.55, 10M/8F)	Chronic	UL (shoulder)	–
Peyre (2020)	France	Non-RCT	I: 12 (55.8 ± 13.5, 9M/3F)<br>C(healthy group): 12 (52.0 ± 6.5, 8M/4F)	Not indicated	LL (CPQ reflex)	–

Sanders (2020)	USA	Within-subject study (cross-over design)	I: 11 (59±12, Not indicated)	Not indicated	UL (hand)	–
Tian (2020)	China	RCT	I: 15 (66.67 ± 13.59, 13M/2F)<br>C: 15 (64.40 ± 13.41,10M/5F)	Not indicated	UL (hand, arm)	–
Choi (2021)	South Korea	RCT	I: 8 (49.88±8.77, 5M/3F)<br>C: 8 (58.50±15.58, 5M/3F)	Not indicated	LL (gait, balance)	–
Donoso Brown (2021)	USA	Single subject design (ABAB)	I: 7 (63.43, 6M/1F)	Chronic	UL	–
Ghai (2021)	Canada	Case study	I: 2 (P1: 66, M, P2: 67, M)	Chronic	UL (hand, arm),	Psychosocial (QoL)
Gonzalez-Hoelling (2021)	Spain	Non-RCT	I: 28 (65.7 ± 12.7, 16M/12F)<br>C: 27 (62.2 ± 8.9, 19M/8F)	Subacute	LL (gait, independence)	–
Haire (2021)	Canada	RCT	I1(TIMP): 10 (54.7± 10.76, 5M/5F)<br>I2(TIMP+cMI): 10 (55.5±15.01, 5M/5F)<br>I3(TIMP+MI): 10 (57.6±11.14, 6M/4F)	Chronic	UL (arm)	–
Raglio (2021)	Italy	RCT	I: 33 (62.4±8.9, 17M/ 16F)<br>C: 32 (64.7±16.0, 18M/14F)	Subacute	UL (finger, hand)	Psychosocial (QoL, pain)
Segura (2021)	Spain	Non-RCT	I: 5 (52.6 ±13.3, 1M/4F)<br>C(healthy group): 20 (65.3 ± 6.85, 10M; 63.5 ± 8.77, 10M/10F)	Chronic	UL(hand, arm)	–
Wang (2021)	China	RCT	I: 30 (61.12±7.49, 8M/22F)<br>C: 30 (61.02±7.51,19M/20F)	Not indicated	LL (gait, balance)	–
Young (2021)	USA	RCT	I: 23 (49.6±13.8, 10M/13F)<br>C: 24 (52.1±9.8, 9M/15F)	Chronic	LL (gait, functional movement)	–
Hankinson (2022)	Australia	RCT	I: 10 (Not indicated, 5M/5F)<br>C: 12 (Not indicated, 8M/4F)	Subacute	UL (hand), LL	–
Kantan (2022)	Denmark	Preliminary usability study	I: 6 (Not indicated, 4M/2F)	Subacute	LL (gait, balance, functional movement)	–
McCue (2022)	UK	Non-RCT (acceptability and deliverability study)	I: 8<br>C: 4<br>(Total-70±11, 5M/7F)	Chronic	LL (gait, balance)	–
Mishr (2022)	India	RCT	I: 10 (59.2±8.216, 5M/5F)<br>C: 10 (52.5±10.99, 8M/2F)	Chronic	UL (hand), LL(gait)	–
Mizuta (2022)	Japan	Cross-sectional study	I: 40 (70.4±10.3, 24M/16F)	Subacute	LL (gait)	–
Ruotsalainen (2022)	Finland	Case study	I: 1 (56, M)	Not indicated	LL, UL	Psychosocial (mood, fatigue)
Shaw (2022)	UK	RCT	I: 30 (71±10, 20M/10F)<br>C: 29 (66±13, 21M/8F)	Not clearly indicated	LL (gait, balance)	–
Collimore (2023)	USA	One-group pre/post study	I: 10 (60.2, 7M/3F)	Chronic	LL (gait)	–
Douglass-Kirk (2023)	UK	One-group pre/post study	I: 20 (53±14, 15M/5F)	Chronic	UL (arm)	–

Kantan (2023)	Denmark	Case study	I:9 (55.4, 5M/4F)	Chronic	LL (gait)	–

Note. C = control group; DMCT = dose-matched conventional treatment; F = female; I = intervention group; LL = lower limb; M = male; mCIMT = modified constraint-induced movement therapy; MP: minimal paresis; NDT = neuro-developmental treatment; ORAS = over ground walking training with rhythmic auditory stimulation; P = patient; QoL = quality of life; RCT = randomized controlled trial; SC = spastic co-contraction; SP =spastic paresis; TRAS = treadmill walking training with rhythmic auditory stimulation; UK = the United Kingdom; UL = upper limb; USA = the United States.
