# Supplementary material for: Reconsidering music in stroke rehabilitation: a scoping review from auditory stimulus to relational process
Source: Front Psychol. 2026 Jun 5;17:1774971. doi: 10.3389/fpsyg.2026.1774971 (PMC13279217; doi:10.3389/fpsyg.2026.1774971)
Supplement: SUPPLEMENTARY Table S2 — Music and sound variable analysis for interventional studies (n = 71), including intervention content, session details, provider involvement, and auditory stimulus characteristics. [file Data_sheet_2.pdf]

Supplementary Material Table S2. Music & sound variable analysis: Interventional studies (*n* = 71)

First author (Year)	Name of intervention	Content of intervention	Length / Session type (Individual or Group)	Settings (hospital, institution, home, community, etc.)	Provider (MT, PT, OT, Using Device etc.)	Music or sound	Use of music or sound:<br>1. Selection (client or researcher)<br>2. Details of music or sound<br>3. Delivery method (live, recorded, computer generated)
Adamovich (2009)	Virtual Piano Trainer	Training on a virtual piano simulation with real-time arm and finger tracking,<br>System provided visual cues (highlighted keys/fingers), auditory feedback (piano tones), and haptic resistance/assistance	90min per day, total 8-9 sessions; individual	Rehab. Research laboratory	Using device (CyberGlove, CyberGrasp, motion tracking, VR software)	Music	1. Researcher-selected (preset scales, 5–10 note sequences, children's songs)<br>2. .wav files of piano tones mapped to virtual keys; successful keystrokes produced realistic piano sounds; sequences formed recognizable songs<br>3. Computer-generated tones through the VR system; real-time auditory feedback linked to finger movements
Altenmüller (2009)	Music-supported therapy (MST)	Participants trained in playing MIDI piano and electronic drum pads with auditory-motor feedback	30 min per session, 15 sessions over 3 weeks; Individual	Neurological rehabilitation hospital (inpatient)	Music-supported therapy specialist, not specified as MT/PT/OT	Music	1. Researcher-selected exercises and songs;<br>2. 8 tones (G-A-B-C-D-E-F-G); simple melodies (children/folk songs, e.g., 'Ode to Joy'); repetitions emphasized for motor<br>3. Live interaction with digital instruments (MIDI-piano, electronic drum pads with piano sounds)
Amengual (2013)	Music-supported therapy (MST)	Exercises progressed stepwise: single tones, sequences of notes, simple children songs.	30 min per session, 20 sessions over 4 weeks; Individual	Hospital(rehabilitation hospital)	Experimenters/therapists trained in MST (not specified)	Music	1. Researcher/therapist designed stepwise program; no patient-selected music<br>2. Music tones produced by MIDI piano<br>3. Live interaction with digital instruments (MIDI-piano and drum pads), instructor demonstrated, then patient repeated.
Beckelhimer (2011)	Computer-based rhythm and timing training utilizing the Interactive Metronome	IM training (hand/foot tapping synchronized with reference tones and guide sounds through Phases 1-6 of IM protocol)<br>Phase1: learn the reference tone<br>Phase 2: the guide sounds<br>Phase 3: timing skills<br>Phase 4: advanced timing skills	60 min (Stretching-IM 30min.-Occupation-based activities 25min), 3 times/ week, for 4 weeks (12 sessions total); Individual	Hospital (Rehab.)	Device+ Researcher (OT graduate students and research assis.)	Sound	1. 1. Researcher-selected<br>2. 2. Metronome(beeps/clicks) for timing, sequencing, and attentional focus<br>3. 3. Computer-generated auditory signals delivered via headphones, with real-time feedback

	(IM) system	Phase 5: focus skills<br>Phase 6: prolonged focus and timing				
Bunketorp-Kall (2017)	Rhythm-and-music therapy (R-MT)	Participants performed coordinated rhythmic movements with hands and feet in response to auditory and visual cues, combined with music listening.	2 sessions a week during 12 weeks; Group	Community and rehabilitation centers	PT + professional musician	Music with rhythmic and auditory cues	1. Researcher/therapist selected<br>2. Rhythmic patterns, coordinated movement tasks with music accompaniment<br>3. Recorded and live music with rhythmic cues (R-MT)
Bunketorp-Kall (2019)	Rhythm and Music-Based Therapy	While listening to music, participants carried out rhythmic- and cognitively-demanding hand and feet movements, by clapping their hands, tapping their hands on their knees or stamping their feet on the floor in time to the beat, in various sequences and combinations and sometimes simultaneously.	2x/week (90 minutes) for 12 weeks; Not clearly reported	Community center	R-MT certified therapist<br>(Not clearly specified)	Music	1. Not specified<br>2. Not specified<br>3. Not specified
Choi (2021)	Multi-Directional Step-Up Training with RAS	As a multi-directional step-up training tool, three step-up boxes with a height of 10 cm, 20 cm, and 10 cm were used, with a 10 height difference between the adjacent boxes. The experimental group was provided rhythmic auditory stimulation (RAS) during multi-directional step-up training	3x/week for 30 minutes for 4 weeks (=12 sessions); Not clearly reported	Clinic	Not clearly specified	Sound	1. Researcher<br>2. Metronome beat from Soundbrenner (Metronome application on a smartphone), During the step-up training, the previously recorded BPM was provided as RAS for 20 minutes through a Bluetooth speaker. As the patient's skill level increased, the BPM was increased by 5 - 10% each week.<br>3. Recorded sound: metronome sound
Chong (2017)	Keyboard playing	Upper body warm-up, Repetitive individuated finger pressing, Sequential, simultaneous key pressing, and melody playing with finger numbered sheets. Focused on repetitive individuated, sequential, and simultaneous movements to improve dexterity.	30m. 12 sessions, twice per week for 6 weeks; Individual	Rehabilitation unit in a university hospital	Experienced MT	Music	1. Therapist-selected exercises, individualized difficulty adjustment<br>2. Simple melodies, scales, repetitive and sequential finger patterns<br>3. Live playing on MIDI keyboard with auditory feedback
Chouhan (2012)	Rhythmic auditory cueing(RAC)	Walked under rhythmic auditory cueing(metronome beats)	2hrs, 3times/wk for 3 wks; Individual	Hospital	PT using metronome	Sound	1. Researcher-selected tempo<br>2. Steady metronome beats at fixed tempos<br>3. Recorded sound: computer-generated metronome delivered via speakers/headphones
Donoso	Preferred	Stroke survivors with hemiparesis and	Total 6 weeks:	Home	OT	Music	1. Selected collaboratively with participants using

Brown (2021)	music listening during home excise	aphasia completed repetitive upper extremity home programs.	two training sessions at their homes on the home program (once a week), 4 weeks home programs (two to three activities to complete 20 repetition blocks for three sets, two times a day, five days a week); Individual		(A preferred music playlist was developed by the music therapist)		aphasia-friendly questionnaire<br>2. One-hour playlists of familiar and enjoyable songs<br>3. Recorded
Fotakopoulos (2018)	Music-based exercise program	A 5 minutes warm-up period of breathing and flexibility exercises followed by the main part of upper and lower body strengthening, balance and co-ordination exercises on sitting and standing position and trunk movements performed at a moderate intensity and a cool-down period of 5-10 minutes of patients holding hands while moving slowly in a circle listening to music.	4 session per week, 45 minutes each session over 6 months; Group activity	Rehabilitation Center	Instructor	Music	1. Researcher<br>2. The music chosen with consideration of their age was experiential/traditional music that constitutes a youth experience deeply imprinted in the long-term memory of elderly patients,<br>3. recorded
Friedman (2014)	MusicGlove (music-based rehabilitation device)	An instrumented glove that requires the user to practice gripping-like movements and thumb-finger opposition to play a highly engaging, music-based, video game.	3x/week for two weeks (one-hour per each session); Individual	Research Center	Using device + PT (superviser)	Music	1. Not clearly reported<br>2. Subjects played twelve songs with the MusicGlove.<br>3. Device generated
Fujioka (2018)	Music-Supported Therapy (MST)	training using an electronic keyboard and a series of eight electronic drum pads; the levels of difficulty were adjusted by changing patterns, speed, and dynamics of movements, as well as distances and heights of the apparatus.	30 hours of training over a 10 weeks; individual	Research lab	OT & MT	Music	1. Researcher<br>2. keyboard & eight electronic drum pads (pitches on a scale from C to C'),<br>3. live
Ghai (2021)	MST (Music-supported therapy)	Supervised piano lessons with auditory feedback through Synthesia & home-based training program without Synthesia	supervised piano lesson: 1 hour session, 3 times per week, over 3 weeks (total 9 sessions) & a biweekly home-	Rehabilitation center & Home	The training clinician<br>(Not clearly specified)	Music	1. Researcher<br>2. Keyboard<br>3. live

			based training program of 30 min per session; individual				
Gonzalez-Hoelling (2021)	Music-based RAS	A general body warming following the rhythm with a metronome (15 min), a main part of the session of music-based RAS exercises (60 min), and closure of relaxation exercises (15 min)	The music-based RAS for 90 min, three times a week ; individual	Hospital	PT & MT	Music	1. Researcher<br>2. Music overlaid with a metronome; The music chosen was from a variety of past and present musical genres, with a marked pulse, 1/4 or 6/8 rhythm, and variation of beats per minute<br>3. No detailed information (the music-based RAS was carried out by a PT & MT)
Grau-Sánchez (2013)	Music-Supported Therapy: MST with TMS evaluation	Training on MIDI-piano (fine motor control) and electronic drum pads (gross motor movements),<br>Exercises included producing single tones, scales, and simple melodies with stepwise complexity	30 min per session x 20 sessions (4 weeks); individual	Hospital<br>(Rehab. setting)	Therapist<br>(Not clearly specified)	Music	1. Researcher/therapist determined<br>2. 8 white piano keys (G–G′) and 8 drum pads programmed with piano sounds; sequences progressed from tones to melodies<br>3. Live (MIDI instruments)
Grau-Sánchez (2017)	Music-supported Therapy (MST)	playing a keyboard and an electronic drum with affected upper extremity; the exercises with the keyboard involved movements of flexion, extension, adduction, and abduction of the fingers and thumb. The drum training, aimed to enhance gross motor function, required movements of flexion, adduction, and abduction of the shoulder as well as its internal and external rotation, Overall, the training was aimed at increasing the range of movement, coordination, and speed.	The treatment periods (MST-1 and MST-2) both consisted of three MST sessions of 1.5 h per week during 4 weeks (24 sessions [36 hr] in total.); individual	Hospital	Therapist	Music	1. Researcher<br>2. keyboard & an electronic drum set of 8 pads with eight consecutive notes (C~C'); playing simple tone sequences and short melodies<br>3. live
Grau-Sánchez (2018)	Music-supported Therapy (MST)	play a keyboard and an electronic drum set with the affected upper extremity following a modular therapy regime with stepwise increase of complexity	20 individual sessions (5 sessions per week, 30 min each); individual	Hospital	OT	Music	1. Researcher<br>2. keyboard & drum<br>3. live
Haire (2021)	TIMP, TIMP+cMT, TIMP+MI	group1(45 minutes TIMP), group2(30 minutes TIMP, 15 minutes metronome-cued motor imagery [TIMP+cMI]), and group3(30 minutes TIMP, 15<br>minutes motor imagery without cues	all groups: 45 min/session, 3 times/week for 3 weeks (total 9 sessions), group1:	University rehabilitation research facility	Neurologic music therapist	Music	1. Researcher<br>2. rhythmic auditory cue and/or metronome cue, & a specially designed programmable digital interactive sound tablet with 32 surface key squares in 4 rows producing musical sounds on touch

		[TIMP+MI]) TIMP exercises were designed to facilitate retraining of functional movement patterns involving proximal and distal control by mapping rhythmically cued movements onto acoustic and digital instruments	45 min TIMP, group2: 30 minutes TIMP +15 minutes cMI, group3: 30 min TIMP +15 min MI; Individual				3. Device (sonification arm training apparatus)
Hankinson (2022)	"GotRhythm" training	A series of participant-specific movement tasks focusing on different affected body parts were selected to match the rehabilitation goals of each patient, The GotRhythm programme consisted of a warm-up, preparatory activities (BPM/movement selection), main activities and cool down	3 times/week for 6 weeks, 20 min per session (total 18 sessions); Individual	hospital	Device + Clinician	Music	1. Researcher<br>2. Metronome, 8-9 individualised music (pt's preference songs) and augmented auditory feedback along with wearable sensors to deliver a personalised rhythmic auditory stimulation training protocol,<br>3. GotRhythm - a mobile phone-based app
Hsu (2012)	Computerized Evaluation and Re-education Biofeedback (CERB) prototype	Participants performed a Pinch-Up-Holding Activity (PUHA) task using a custom pinch device (thumb + index finger) Biofeedback training: training using the computerized evaluation and re-education biofeedback, Sound was used a s part of the feedback system	35–40 min per session (25 min. conventional + 10–15 min. CERB), 3 times per week × 4 wks (12 sessions total); Individual	Hospital (Rehab. setting)	Device+ OT	Sound	1. Researcher/therapist defined<br>2. Auditory signals (beeps) triggered when pinch force exceeded target range<br>3. Computer-generated auditory cues delivered via the CERB system
Hutchinson (2020)	Music-based digital therapeutic (Automated RAS)	Automated music-based gait training with real-time sensor feedback adjusting music tempo to entrain walking cadence. Progressive and individualized training using closed-loop algorithms.	One session and two additional training visits (30 min each); individual	University clinic; feasibility for home/community use	Device + PT/research staff	Music	1. Researcher-curated familiar genres(ie, classic rock, Motown, or pop), with 4/4 meter; idividualized playlists<br>2. Music tempo adapted 5% increments based on entrainment; overlaid metronome when needed<br>3. Device generated
Jun (2013)	Music and movement therapy (MMT)	The MMT consisted of 3 phases: (1) preparatory activities - a brief stretching with quiet meditational music and sang songs, (2) main activities- sang songs and motions of the healthy side and playing instruments (tambourines, maracas), (3) finishing activities - expression of their feelings and sharing of their experiences.	3 times/week, 1 hour per session, over 8 weeks; Group	Hospital	Researcher + MT	Music	1. Researcher and MT<br>2. Quiet meditational music for BG and songs that were popular during the pt's younger days, playing instruments<br>3. recorded and live
Kim (2011)	Visual and	The auditory step rhythm incorporated into	10-12 min, 4 days;	Stroke	Not	Sound	1. Researcher-selected

	Kinesthetic Locomotor Imagery Training with Auditory Step Rhythm	each of the two types of locomotor imagery training was a constant metronome beat for 30 seconds, which was individually adjusted according to the walking speed of each subject. It was included in the audiotaped instructions for the locomotor imagination phase of each locomotor imagery training.	Individual	rehabilitation center	explicitly mentioned		2. Metronome (adjusted to individual's walking speed)<br>3. Recorded audio
Kim, J. H. (2012)	Rhythmic Auditory Stimulation (RAS)	RAS gait training using smartphone metronome app,<br>5 progressive stages: (1) Forward, backward, and side walking (2) Sit-to-stand (3) Obstacle crossing (4) Stair ascent/descent (5) Forward walking with cadence increased +5% and gradual fading of metronome cues	30min. Training sessions/3times per wks/5wks (15 session total); Individual	Rehab. Hospital	PT	Sound	1. Researcher/ therapist determined (tempo set to patients' comfortable cadence; +5% for final stage)<br>2. Smartphone app, auditory beeps; beats matched to gait cadence, modulated for task difficulty<br>3. Recorded sound: smartphone-generated auditory signals delivered via individual earphones
Kim, J. S. (2012)	Home-based auditory stimulation training	The subjects walked over the ground while listening to a metronome beat. The metronome beat began at 20 pulses per minute, and was incremented by 20 pulses per minute every 2 minutes.	3x/week for 6 weeks (10 min in each session); Individual	Home	Not clearly reported	Sound	1. Researcher<br>2. The metronome beat could be listened to as a constant beat for the whole 10 minutes<br>3. Recorded sound, Materials for delivery are not clearly reported.
Kirk (2016)	Digital Musical Instruments (DMIs)	'drumming' to the beat of self-chosen tunes using bespoke digital drum pads	30 min, 3 per week, total 15 sessions for 5 weeks; individual	Home	Researcher + using device (iPad + drum pads)	Music	1.Participant<br>2. Participants' favorite songs, and 4 percussion sounds(Hi-Hat, Woodblock, Snare and To,) were mapped to digital drum pads<br>3. Device generated
Lee, S. (2018)	Gait training with bilateral rhythmic auditory stimulation (GTBR)	The training consisted of warm-up for 5 min, gait training for 20 min and cool-down for 5 min. During the gait training, the auditory stimulation sound was set for each individual to be used. While subjects walked, each sound was adjusted to the heel strike of each foot.	30 min a day, 5 days a week, for 6 weeks, in addition to conventional rehabilitation; Individual	Rehabilitation center	PT	Sound	1. Researcher-selected based on individual gait analysis<br>2. Metronome beat (wireless headphone)<br>a. Different pitch sounds to distinguish sounds applied to both legs<br>b. Auditory stimulation increased by 10% for paretic side and 5% for non-paretic side compared to comfortable walking speed<br>3. Recorded sound using digital audio editing software
Luft (2004)	Repetitive Bilateral Arm	Repetitive bilateral forward–backward arm movements (in-phase and anti-phase)	18 sessions total, 3sessions per	Baltimore Veterans	Rehab. Therapist	Sound	1. Researcher determined (pacing set to participant's comfortable rhythm at baseline, maintained

	Training with Rhythmic Auditory Cueing (BATRAC)		week/1hr/6weeks; Individual	Affairs Medical Center (Outpatient rehab.)			throughout training)<br>2. Simple metronome beeps providing rhythmic timing cues for bilateral coordination<br>3. Recorded sound: Computer/metronome-generated auditory signals via speakers
Mainka (2018)	RAS and Treadmill Training	Patients listened to functional training music while walking on the treadmill.<br>The beat rate of the music was set to match the patients' cadence on the treadmill. Once audio-motor coupling was achieved, the musical tempo was slowed down a little in order to induce greater step lengths.	5 times a week for 4 weeks (15-20 min in each session);<br>Individual	Clinic	Physiotherapist<br>(continually supervised by a certified neurologic music therapist)	Music	1. Researcher<br>2. clearly structured rhythm with strongly accentuated meter, familiar melody, no lyrics, and a superimposed salient high-pitch bell sound<br>3. Recorded: via ear plugs through an ordinary MP3 player
Malcolm (2009)	Rhythmic Auditory Stimulation (RAS) upper limb training	Arm reaching with auditory cues, A combination of on-site and home-based training. The training procedure and structure of skill practice during RAS were designed to incorporate the following variables: movement timing, range of motion, and feedforward processing.	2-week (onsite + home-based)<br>(1) Monday, Wednesday, and Friday: 1 hour/day of onsite training and 2 hours of home-based training, (2) Tuesday and Thursday: 3 hours of home-based training; individual	Hospital and outpatient therapy centers, home	OT	Sound	1. Researcher-selected<br>2. Metronome<br>3. Recorded cue
McCue (2022)	Auditory rhythmical cueing	Auditory rhythmical cueing was provided for a total of 10 different home and outdoor gait and balance exercises. Exercises were gradually progressed according to patient ability by increasing the speed/intensity, duration or amount.	3x/week for 6 weeks (30 min in each session): supervised session - once per week, self-managed sessions -two per week; Individual	Home and outdoors	PT or Researcher (superviser)	Sound	1. Researcher<br>2. A single tone of metronome. During the supervised sessions, the frequency for the auditory cue during each exercise was selected by the study provider<br>3. Recorded sound, Materials for delivery: a commercially available metronome or a free metronome app for a mobile phone
Mishr (2022)	Resisted bimanual therapy with rhythmic	Resisted bimanual therapy with rhythmic auditory cueing group received calibrated weight cuff of 912g that was tied over the non-paretic forearm.	15 sessions within 3 weeks, (45-60 min per session); Not clearly reported	Tertiary health care center	Therapist	Sound	1. Researcher<br>2. Metronome was used as an auditory cue that was rhythmic in nature with a beat frequency of 20 beats

	auditory cues	Bimanual movement task included: 1) Transfer of the ball from one side of vertical separator to another side, 2) Transfer and passing the ball to therapist from one side to other using trunk rotation in a circular manner, 3) Controlled rolling of the ball on the wedge, 4) Controlled rolling a towel (cleaning of the wall) in up to down, side to side and circular movement and 5) Movement of the ball in Proprioceptive Neuromuscular Facilitation(PNF) diagonal chopping and lifting pattern					per minute<br>3. Recorded sound: Metronome
Muto (2012)	Interactive cueing with Walk-Mate	Walk-Mate generates a model gait rhythm in response to the user's locomotion in real-time, and presents it using auditory stimuli. The subjects were requested to focus on the rhythmic sounds, and coordinate their leg motion with it while walking.	Twice a day for 5 consecutive days: 9 training sessions (In the first trial on the first day, only the initial gait condition was measured); Individual	Not clearly reported(a quiet room)	Using device + Researcher	Sound	1. Researcher<br>2. Rhythmic sound, the sounds presented may use different tones on the left and right<br>3. Computer generated: The sound was presented via wireless headphones.
Nikmaram (2019)	Musical Sonification	Playing several simple nursery rhymes or other familiar tunes only by moving their affected arm in the three-dimensional sonification space.	Regular training sessions lasted approximately 30 min.<br>Site1: Number of training days , median (range): Treatment 15 (11-15); Site2: Number of training days , median (range): Treatment 22 (7-40); Individual	Clinic	Using device	Music	1. Researcher-designed based on movement parameters<br>2. Details of music: a musical note-pitch name of the C major scale from c' (at the bottom) to a' (top)<br>a. Qualitative sound changes when hand moved relative to the frame/body<br>b. Sound mapped to three-dimensional movement space<br>3. Computer generated in real-time based on movement capture
Park (2015)	TRAS (Treadmill + RAS), ORAS (Overground + RAS)	Compared treadmill walking with RAS (TRAS) vs. overground walking with RAS (ORAS).	30 min sessions, 5 per week for 3 weeks; Individual	Hospital Rehabilitation center	PT	Sound	1. Researcher-selected tempo<br>2. Computer-based metronome synchronized to treadmill/overground walking<br>3. Recorded sound: metronome sounds via wireless

							headset.
Park (2018)	Auditory Stimulation Robot-assisted Gait Training (ARGT)	ARGT performed gait training using the rehabilitation robot. ARGT is a method of adjusting the walking speed to the regular rhythm of the metronome producing the auditory signal. In order to control the movement of progressive intensity, the rhythm of the metronome was adjusted at the speed increased by 5% walking speed was intervened.	6 weeks, 3 times a week, for 45 minutes each; individual	Not clearly specified	PT	Sound	1. Researcher<br>2. Metronome beat(according to initial patient's cadence)<br>3. Recorded sound: Metronome
Raghavan (2016)	Music Upper Limb Therapy-Integrated (MULT-I)	group music-making (playing instruments) intervention for upper limb:<br>(1) Introduction: OT led musically supported stretches of the trunk and upper limb and a focus group discussion, (2) Music making - improvised live music (instrumental playing), (3) wrap-up for group discussion	45-min, twice a week for 6 weeks; Group	Hospital	MT + OT	Music	1. MT & participants<br>2. Improvised live music (instrumental playing-drums, bells, shakers, mallets, chimes, piano and harp) and MT provided musical support to reflect the effort and expression of the subjects by adjusting the accompaniment including dissonance melodies, high melodic registers and the style of music<br>3. Live
Raglio (2017)	Relational active music therapy approach (RAMT)	free interactions between patient and music therapist using rhythmical-melodic instruments in a non-verbal setting. During RAMT, MT invites patients to play an active role and to interact using musical instruments.	20 RAMT sessions; 30 minutes per session, 3 times a week; individual	Hospital	MT	Music	1. MT and patient<br>2. rhythmic and melodic instrument(xylophone, glockenspiels, drums, bongos, ethnic percussion<br>3. live
Raglio (2021)	Sonichand (musical sonification)	music-based sonification approach<br>(training of pronation and supination of the forearm, ulnar and radial deviation of the wrist and hand grasping movements)	5 days/week for 4 weeks, (a total of 20 sessions); 35 min per session; individual	Rehabilitation Center	Using device + therapist	Music	1. Researcher<br>2. Synthesized sounds/musical texture and their parameters (mainly rhythm, pitch/melody, intensity/dynamics, harmony and timbre) & flute, cello, guitar, piano etc., as well as some timbre variants for the pad performed by a synthesizer,<br>3. Device generated
Richard (2008)	modified bilateral arm training	a modified form of BATRAC (modBATRAC)<br>Bilateral forward–backward reaching/pulling	18hrs. total, Four 2.25hrs per week for 2weeks; Individual	a clinical setting at the University of	Not clearly specified	Sound	1. 1. Researcher-determined (self-selected daily speed, but beats generated by metronome)<br>2. 2. Metronome pulses, adjusted for participant's

	protocol (modBATRAC)	arm movements, alternating between in-phase and anti-phase		Maryland			training speed; phase alternation (in-phase vs anti-phase)<br>3. 3. Recorded sound: Computer/metronome-generated auditory signals, delivered via speakers
Ripollés (2016)	Music-supported therapy (MST)	instrumental playing: digital keyboard, electronic drum set comprising 8 pads: tasks involved playing simple musical patterns(from single tones to parts of songs) with the affected hand	20 sessions of 30 min over a 4 week period; individual	Hospital	Therapist	Music	1. Researcher<br>2. Simple, structured melodic and rhythmic patterns, popular Spanish music<br>3. Live
Ruotsalainen (2022)	Combining MT techniques with physiotherapy techniques	(1) Opening discussion<br>(2) Whole body tasks -Rhythmic exercises with music (e.g., rhythmic walking, standing, sitting, clapping hands, tapping knees, drumming with hands or mallets, stompling feets, etc.), (3) Upper body tasks - clapping hands, tapping knees, drumming, (4) Relaxation - listened to BG while sitting or lying down, focusing on breathing and reducing bodily tension Guided Imagery techniques were used.	10 sessions; once a week; approximately 40 min per session; individual	Private physiotherapy clinic	MT	Music	1. Researcher and patient<br>2. musical stimuli consisted of the patient's own chanting of a chosen rhyme, the therapist's singing, or pre-recorded music of patient's preference and background music for relaxation,<br>3. Live and recorded
Sanders (2020)	MusicGlove	Wearable sensor-based game for finger rehabilitation.<br>Subjects were free to modulate the difficulty of their MusicGlove training by changing the number of grip types needed to play, and/or by selecting songs at three different difficulty levels.	4.1 (+/− 3.2 SD) hours, which was 46% of the recommended 9 hours (at least three hours of their intervention per week for 3 weeks); Individual sessions (home-based)	Home	Using device + Initial training by a rehabilitation therapist	Music	1. Therapist and subjects<br>2. Songs at three different difficulty levels, difficulty was determined by the number of target notes per minute of song.<br>3. Computer generated through the MusicGlove game/tablet interface
Schauer (2003)	Musical motor feedback (MMF)	The test group practised walking with the musical motor feedback (MMF). The MMF device consists of sensor insoles that detect the ground contact of the heels, and a portable music player compatible with the MIDI standard. The portable MMF device was fixed to the patient's belt and thin wires	5 days per week for 3 weeks (20 minutes each day); Individual	Inpatient rehabilitation hospital	Using device + verbally encouraged by therapists	Music	1. Not clearly reported<br>2. The music was played at an adjustable speed, which was estimated from the time interval between two consecutive heel-strikes. The required time period to play a quarter meter was stretched or compressed instantly to coincide with the patient's present step duration.

		led to the insoles					3. Device generated: The music was presented via plugged headphones.
Schneider (2007)	Music-Supported Training (MST)	Stroke patients trained on MIDI piano and electronic drum pads to improve upper limb motor recovery. Training progressed stepwise from simple tones to melodies;	30 min each, 15 sessions over 3 weeks; Individual	Neurological rehabilitation hospital	Not specified. Therapists delivered program	Music	1. Researcher-designed stepwise program, no patient choice<br>2. Children's or folk songs: Twenty different songs were available for the eight tones (e.g., Ode to Joy).<br>3. Live
Schneider (2010)	Music supported training(MST)	Patients trained on MIDI-piano (fine motor) and electronic drum pads (gross motor), Stepwise modular training (10 levels) progressing from simple tone repetitions to scales and short melodies	15sessions for 3weeks/ 30min; Individual	Neurological Rehabilitation hospital	Researcher (Not clearly specified)	Music	1. Researcher-selected (fixed repertoire of 20 songs; therapist guided progression)<br>2. Piano tones from MIDI keyboard or drum pads; progression from isolated notes to simple songs with strong rhythmic and melodic structure<br>3. Live
Scholz (2016)	Musical sonification training	arm movements that were sonified, or triggered musical sounds, in real time, ex.Vertical movements in a three-dimensional sonification space resulted in a change in brightness of sound	30 min/day for 10 days; individual	Hospital	Using device	Music	1. Researcher<br>2. Familiar melodies and harmonic tones<br>3. Device-generated (a three-dimensional sonification space)
Segura (2021)	A home-based enriched Music-supported Therapy (eMST)	1) playing percussion instruments to warm up the affected upper limb and train gross motor skills: patient was asked to play rhythmic pattern with a specific tempo (Level 1-8) and 2) playing the MIDI-piano to train fine motor skills: patient played different note sequences on MIDI-piano using different fingers or combination of fingers with the affected hand (Level 1-9)	approximately 1 hour, 3 times/week for 10 week (total 30 sessions); Individual	Hospital & home & remote	MT + App (on an electronic tablet)	Music	1. Researcher<br>2. playing with the affected limb rhythmic patterns with percussion instruments (tambourine, djembe, marcas, egg shaker, rainstick, castanets, guiro), & a MIDI-piano keyboard exercises consisted of playing white key notes<br>3. Live
Shaine (2014)	bilateral arm training with rhythmic auditory cueing (BATRAC)	The BATRAC protocol uses a device that provides assistance to the paretic upper extremity and provides both inphase (symmetrical) and antiphase (asymmetrical) movement training accompanied by rhythmic auditory cueing.	1 hour/session, 8 weeks, 3x/week; individual	Hospital clinic	PT	Sound	1. researcher Set at the patient's preferred speed;<br>2. Not specified; Auditory cueing was set at the patient's preferred speed; this was established at the fi rst session by asking the patient to assume a comfortable speed that he/she could continue for 5 min (frequencies ranged from 0.25 to 1.0/s).<br>3. Not specified
Shaw (2022)	Auditory rhythmical	Auditory rhythmical cueing was provided for 10 gait and balance exercises which were	3x/week for 6 weeks (30 min in each	Home and outdoors	Therapist or Researcher	Sound	1. Researcher<br>2. A single tone was used and the cueing frequency

	cueing	gradually progressed according to participant ability. Progression included increasing cueing frequency, increasing number of repetitions or time spent on an exercise and increasing task difficulty such as increasing number of turns.	session), one session per week was supervised face-to-face by a trained member of the research team and two sessions were self-managed. ; Individual		(superviser)		was dependent on exercise type<br>3. Recorded sound, Materials for delivery: A commercially available metronome or a free metronome app for a mobile phone
Shin (2015)	Rythmic auditory stimulation (RAS)	(1) A participant walked along a 10m flat walkway three times without RAS. Walking cadence was calculated. (2) The identified initial tempo signaled by metronome beats was set to the participant's cadence. (3) A participant walked with RAS after an adaptation step that included finger-tap. (4) The final 1–2 minutes was spent by fading out the rhythmic stimulation to monitor the independent carryover effect.	3x/week for 4 weeks (30 min per each session); Individual	Clinic	Music therapist	Music	1. Researcher<br>2. A live rhythmic pattern was provided using a composed four-chord progression with metronome beats on a keyboard.<br>3. Live
Silveira (2018)	iPad based intervention	1) iPad based instrument using the app. ThumbJam combining a FES<br>(improvisation playing)<br>2) Independent practice with keyboard	45 minute session with music therapist (once a week for 5 wks), Independent practice with keyboard , 7 months follow up	hospital	Registered Music Therapist<br>(OT provided FES)	Music	1. Improvisation by client<br>2. Improvisation on ThumbJam and keyboard, song learning<br>3. Live
Song (2015)	Bilateral training with rhythmic auditory cueing (BATRAC)	Performed repetitive bilateral arm movements with rhythmic auditory cueing	30 minutes/day, 5 times/week, for 12 weeks; individual	Not clearly specified	PT	Sound	1. researcher<br>2. simple rhythmic beats (not harmonic or melodic music)<br>3. Not clearly specified
Street (2018)	TIMP	TIMP: instrument and iPad playing for distal motor exercises with live guitar accompaniment	2x/week, 6 weeks<br>(20–30 minutes in each session); Individual	Home	Trained neurologic music therapist	Music	1. Researcher<br>2. Strongly pulsed facilitating patterns, tempo matched to participant<br>3. Live guitar, iPad apps
Street (2019)	TIMP	1. TIMP: upper limb extension and flexion	2x/week for 6 weeks	Home	Neurologic	Music	1. Researcher-adjusted parameters (e.g., adding

		using musical instruments (cymbal)<br>2. TIMP: Thumb extension/flexion using touchscreen guitar (Garageband app on iPad)<br>Exercises targeted at upper limb rehabilitation including grip, lift, and, combined movements	(20-30 min in each session); Individual		music therapist		crescendo to improve synchronization)<br>2. Music: Strumming of chords with adjustable intensity/crescendo, Virtual guitar strings producing sound with thumb movements, Musical cues for priming and timing of movement<br>3. Live (cymbal playing) and Computer generated (iPad Garageband app)
Suh (2014)	RAS	The RAS training was administered in four steps of gait training. The first step consisted of a 5 meters warm up walk, a 3 minutes-rest, followed by a 10 meterwalk depending on a patient's endurance level without rhythm cue. The second step of gait training consisted of the 2 minutes-rest, 1 minute toe tapping, followed by 10 meter- walk with the rhythm cue provided in the tempo of the participant's cadence. The third and fourth step of gait trainings were executed in similar format except the cadence was provided with an increase of 5% and 10% respectively for each step.	RAS - 15 min 5 per week for 3 weeks; Individual	Hospital	PT	Sound	1. Therapist-selected<br>2. The rhythmic stimulus in the training session was provided using a digital Musical Instrument Digital Interface (MIDI) software. The rhythm stimulation was composed of single tone series in 4/4 time signature.<br>3. Recorded sound (MIDI)
Thaut (1997)	Rhythmic auditory stimulation (RAS)	(1) After an initial cadence assessment during a 1 to 2 min warm-up walk, the rhythm frequency was matched to the gait cadence for the first quarter of the session. (2) During the second and third quarter the rhythm frequency was incrementally increased by 5 to 10%, depending on the patient's ability. (3) The last quarter was spent with RAS intermittently faded to train for independent carry-over of improved gait patterns.	twice daily, 5 days/week for 6 weeks (30 min each) ; Individual	Research center or hospital	PT	Music	1. Researcher<br>2. Instrumental music in 4 different styles was prepared (classic, folk, country, jazz). The music was recorded in 2/4 meter to match the rhythm of the step patterns in gait. A metronome beat was overlaid on the strong beat of the music to enhance the rhythmic perception for the patient.<br>3. Pre-recorded: The music was played over headsets that were prerecorded on a synthesizer /sequencer module.
Thaut (2007)	Rhythmic Auditory Stimulation (RAS)	walking to rhythmic auditory cues (e.g., metronome beats or music).<br>Steps: (1) walk with cue frequencies matched to the gait cadence, (2) walk with cue frequencies increased in 5% increments,	30 minutes, 5 times per week, for 3 weeks; individual	Research center or hospital	Not clearly specified	Sound, music	1. researcher (according to participants' cadence)<br>2. metronome sound/rhythmic music<br>3. prerecorded, computer-based

		(3) trained for adaptive gait patterns, for example, ramp or step walking, were practiced. (4) walk with fading cues					
Tian (2020)	RAS for upper extremity	The RAS therapy was performed by practicing movements(e.g., shoulder flexion/extension, elbow flexion/extension, forearm pronation/supination, etc.) of certain tasks with auditory cues at a gradually increased rhythm with or without instruments.<br>Participants received physical therapy, occupational therapy, and RAS	30 min physical therapy & 30 min occupational therapy, 5 days/week for 4 weeks + 30 min RAS everyday for 4 weeks; Individual	hospital	Not clearly specified	Sound	1. Researcher<br>2. rhythmic auditory cue by metronome,<br>3. Recorded sound : Metronome software
Tong (2015)	Music-supported therapy (MST)	instrumental playing: keyboard, percussion.<br>Steps: (1) beating the same keyboard 60 times within 1 minute; (2) beating the eight keyboards one-by-one 60 times within 1 minute; (3) beating the eight keyboards in a specified random order 60 times within 1 minute; (4) playing a piece of simple melody.	20 sessions, over 4 weeks 45 min; individual	Rehabilitation Research Center	Not clearly specified	Music	1. researcher<br>2. simple familiar melodies, ‘‘Are you sleeping’’, ‘‘Edelweiss’’, and ‘‘Auld lang syne”<br>3. Live (A. wooden percussion instrument, B. aluminum sheet lyre, C. percussion instruments (soft sponge), D. aluminum sheet lyre (soft sponge))
Van Delden (2013)	mBATRAC	mBATRAC; Bilateral rhythmic flexion/extension of wrist and fingers paced by auditory metronome beats	60 min/session, 3 sessions per week, for 6 consecutive weeks (18 sessions); Individual	rehabilitation center	Not clearly specified	Sound	1. Researcher/ therapist selected<br>2. Simple rhythmic metronome beeps to cue bilateral wrist/finger flexion–extension movements<br>3. Recorded sound: Computer/metronome-generated auditory signals
Van Vugt (2014)	Music-Supported Motor Training (MSMT)	playing simple finger exercises and familiar children’s songs on the piano<br>Steps: (1) played simple finger exercises such as a five-tone scale up and down and other patterns with their paretic hand (2) learned to play one from a set of simple children’s songs (3) learn additional songs from the set prepared by the therapist.	30 min for 3-4 weeks (10 sessions); individual & group(pair)	Hospital	Therapist	Music	1. Therapist<br>2. simple familiar children song<br>3. live (digital piano)
Villeneuve (2013)	Music-supported therapy(MST)	Piano training: Musical tasks involved use of all five fingers of the affected hand,<br>Training emphasized accuracy, timing, and	3-week structured piano training(9 supervised lessons, 3×/week, 1 hour	Home	Not clearly specified	Music	1. Researcher-defined<br>2. electronic piano tones, structured songs: nine musical pieces in progressive difficulty: (1) simple

		speed; tempo increased stepwise (30 → 60 bpm) when >80% accuracy achieved	each)<br>Complemented with home piano practice (biweekly, ~30–50 min per session); individual				consecutive finger sequences, (2) intermediate intervals, (3) complex chords<br>3. Live
Villeneuve (2014)	Piano training program	Steps: (1) involved movements of consecutive fingers [e.g., digit 1–2–3–4–5], (2) involved third, fourth, and fifth intervals or movements of non-consecutive fingers [e.g., 1–3–5–2–4], (3) involved chords, that is two fingers played at the same time.<br>+ the tempo was increased by steps of 10%	60 min 3 sessions per week for 3-weeks (total 9 sessions) + a biweekly home program (30 min/session); Individual	Home	Therapist<br>(rehabilitation therapist without musical background)	Music	1. Researcher<br>2. Pieces were composed by an experienced musician and were designed to be musically pleasant based on simple harmonic rules of composition as well as of relatively short duration and easy to remember<br>3. live (MIDI piano)
Wang (2021)	Music therapy _ Routine rehab.	Patients walked three times daily for 1 h: first metronome adjusted to walking velocity, then therapist played familiar melodies at that rhythm; at the end, metronome re-measured walking velocity for next session.	One hour each with 3 min rest every 10 min, 3 times daily for 4 weeks; Individual	Hospital	Therapist played familiar melodies; not specified discipline	Music + Sound	1. Therapist-selected familiar melodies; tempo guided by metronome<br>2. Metronome used to measure/ adjust cadence; familiar melodies used to guide walking rhythm<br>3. Live therapist-performed music; metronome device
Whitall (2000)	Repetitive Bilateral Arm Training with Rhythmic Auditory Cueing (BATRAC)	BATRAC: bilateral T-bar arm trainer; repetitive forward–backward arm movements,<br>Movements cued by auditory metronome beats at participant's preferred speed	3times per week x 6 wks (18 sessions total)<br>20 min active training per session (within a 1-hour therapy session including rests); Individual	Medical Center and Rehabilitation Center	Not clearly specified	Sound	1. Researcher/ therapist determined (individual's preferred pace established at baseline)<br>2. Simple auditory metronome beats<br>3. Recorded sound: Computer/metronome-generated beats, delivered via speakers in training room
Whitall (2011)	Bilateral Arm Training with Rhythmic Auditory Cueing (BATRAC)	bilateral T-bar arm trainer; repetitive forward–backward arm movements	1 hour/session, 3 sessions/week, 18sessions for 6 wks;<br>Individual	Hospital<br>(Rehab. Clinic)	Not clearly specified	Sound	1. Researcher/therapist determined (paced at participant's preferred rhythm; constant frequency after session 3)<br>2. Simple metronome beeps cuing bilateral in-phase and anti-phase arm movements<br>3. Recorded sound: Computer/metronome-generated auditory cues via speakers

Wright (2017)	A Home-Based Auditory-Cued Step Training	walking with music and metronome beat	15 min on 5 days/week; two 3 week-block; Individual	Home	Researcher	Music	1. Researcher,<br>2. Popular music and metronome (music overlaid with an auditory metronome)<br>3. Recorded: mp3 player with either speaker or headphone
Young (2021)	Movement-to-music (M2M) intervention	Various movement combinations choreographed to music were used. Each session began with 2 seated warm-up routines. It then transitioned to an upper extremity muscle strengthening routine performed in a seated position, followed by cardiorespiratory endurance, lower extremity muscle strengthening, and balance routines.	3x/week for 12 weeks (60 min in each session) ; Not clearly reported	Community-based fitness facility	Trained dance instructors	Music	1. Not clearly reported<br>2. A wide range of tempos is used: The slower tempos for bigger movements and the faster tempo for smaller movements (bpm specified)<br>3. Not clearly reported
Zondervan (2016)	MusicGlove	Used a sensorized glove connected to a music-based video game; performed gripping movements to play notes in rhythm with songs	at least three sessions per week, for three consecutive weeks, for a total of 9 h of therapy; individual	Home	Using device	Music	1. Researcher<br>2. popular song<br>3. computer-generated (MusicGlove)

Note. BPM = beats per minute; FES = functional electrical stimulation; MT = music therapist; OT = occupational therapist; PT = physical therapist; RAS = rhythmic auditory stimulation; TIMP = therapeutic instrumental music performance; TMS = transcranial magnetic stimulation; VR = virtual reality.
