# Supplementary material for: Reconsidering music in stroke rehabilitation: a scoping review from auditory stimulus to relational process
Source: Front Psychol. 2026 Jun 5;17:1774971. doi: 10.3389/fpsyg.2026.1774971 (PMC13279217; doi:10.3389/fpsyg.2026.1774971)
Supplement: SUPPLEMENTARY Table S3 — Music and sound variable analysis for non-interventional studies (n = 26), including task content, session details, provider involvement, and auditory stimulus characteristics. [file Data_sheet_3.pdf]

Supplementary Material Table S3. Music & sound variable analysis: Non-interventional studies (n = 26)

First author (Year)	Name of non-intervention	Content of Non-intervention	Length / Session type (Individual or Group)	Settings (Hospital, institution, home, community, etc.)	Provider (MT, PT, OT, Using Device etc.)	Music or Sound	Use of Music or sound: 1. Selection (client or researcher) 2. Details of music or sound 3. Delivery method (live, recorded, computer generated)
Aluru (2014)	No auditory cueing, happy sounds, self-selected music, and metronome beat	Bimanual-to-unimanual training for the paretic side of wrist movement in 4 different auditory stimuli conditions:<br>1) no auditory cueing; 2) non-musical happy sound; 3) self-selected music; 4) metronome beat	One-time session, each condition consisted of 18 15-s trials of wrist flexion and extension, where subjects performed two bimanual trials followed by one unimanual trial with the paretic hand;<br>Individual session	Research Laboratory	Using wrist trainer device & research staff	Music & sound	1.a. Self-selected for the up-tempo major key song (chosen during visit 1)<br>b. Researcher-selected for happy sounds and metronome<br>c. Metronome set at each individual's comfortable tempo<br>2. a. Happy sound: baby's laughter recorded for 11 seconds and looped continuously<br>b. Music: up-tempo major key song<br>c. Metronome: beat set at individual's comfortable tempo<br>3. a. Recorded (happy sounds- looped recording)<br>b. Recorded (self-selected music)<br>c. Recorded
Cha (2014)	Rhythmic Auditory Stimulation (RAS)	RAS under five conditions:<br>(1) no RAS (baseline),<br>(2) baseline-matched tempo (0%),<br>(3) −10% of baseline, (4) +10% of baseline, and (5) +20% of baseline. Participants listened to the beat for 30 seconds before walking, then synchronized steps with the rhythm.	One-time experimental session (< 60 min total);<br>Individual session	Rehabilitation center	Not clearly reported<br>(author: PT)	Sound	1. Researcher-selected<br>2. Metronome beat<br>3. Recorded
Chen (2016)	Augmented auditory feedback	Arm reaching task in 3 different types of augmented auditory feedback: (1) real-time auditory feedback reflecting movement quality (2) rhythmic cueing to guide movement timing, and (3) no	Short-term practice, arm reaching task with each condition on separate day (1 day, 72 trials);<br>Individual session	Hospital	Using device (computer, goniometer sensors, headphone)	Sound	1. Researcher-selected<br>2. Consonant/dissonant sound for real-time auditory feedback, metronome sound for rhythmic cueing,<br>3. Computer-generated

		feedback					
Collimore (2023)	Autonomous control of music to retrain walking	Music-based gait training system where participants walked with adaptive music feedback controlled by a closed-loop system.	One-time session (30 minutes); Individual session	Rehabilitation laboratory	Automated device-based system (digital therapeutic prototype)	Music	1. Researcher-selected<br>2. Songs screened for consistent beat saliency and rhythmic stability, and the playlist spanned various tempos and musical genres. Familiar songs adapted to cadence; tempo adjusted in real time.<br>3. Computer-generated
Crosby (2020)	Rhythmic Auditory Stimulation (RAS)	Participants with post-stroke temporal gait asymmetry walked with and without metronome beats.	One-time session-lab study; Individual session	Rehabilitation Institute	Research staff	Music & Sound	1. Researcher-selected<br>2. Simple metronome tones<br>3. Recorded
Douglass-Kirk (2023)	Real-time auditory feedback	An automated digital approach for identifying and signaling abnormal movements in upper extremity using real-time auditory feedback. The music was either on or off (muted) according to whether the system detected abnormal movements during an active forward reaching task.	One-time session, 50 repetitions of an active reaching task while listening to self-selected music with auditory feedback to signal when abnormal movements occurred; Individual session	Rehabilitation laboratory	Using device & PT or OT	Music	1. Patient-selected<br>2. Favorite piece of music which matched their baseline movement tempo<br>3. Recorded
Ford (2007)	Step to the beat (condition 1) and move their arms and legs to the beat (condition 2)	Two instruction conditions: 1) "step to the beat" and 2) "move arms and legs to the beat" with metronome frequencies at 1.0, 1.2, 1.4, 1.6, and 1.8 Hz	One-time session; Individual session	Clinical movement science laboratory	Not clearly reported	Sound	1. Researcher-selected<br>2. Metronome frequencies at 1.0, 1.2, 1.4, 1.6, and 1.8 Hz<br>3. Recorded
Jung (2012)	Integrated visual and auditory stimulus	Subjects walked while looking at a screen and listening to auditory signals produced by a metronome. The speeds of the visual and auditory stimuli were divided into 3 levels: 50%, 100%, 150% the walking speed of each patient.	One-time session, each (50%, 100%, 150%) was performed 3 times, and 3 min of break time was given to patients between measurements; Individual session	Hospital	Not clearly reported	Sound	1. Researcher-selected<br>2. Auditory signals produced by a metronome<br>3. Recorded

Kang (2020)	Rhythmic Auditory Cueing (RAC) and Melodic Auditory Cueing (MAC)	No auditory cueing (NAC), Rhythmic Auditory Cueing (RAC), Melodic Auditory Cueing (MAC) / Five trials, each consisting of abduction, holding, and adduction phases, were blocked with one of the three cueing conditions and randomly presented to the participant while avoiding the consecutive presentation of the same cueing condition	Three blocks of three cueing conditions (NAC, RAC, and MAC); Individual session	Rehabilitation center	Not clearly reported	Music & Sound	1. Researcher-selected<br>2. MAC utilized "pitch-related elements" including "ascending and descending melodic contours" to cue upward and downward movements; Music elements included "rhythmic, melodic, and harmonic elements to provide temporal, spatial, and dynamic information about the movement"<br>3. Recorded
Kantan (2022)	Musical biofeedback	Digital musical interactions for balance, sit-to-stand, and gait training. The framework comprises wireless wearable inertial sensors and software. The software enables layered and adjustable music synthesis and has a generic movement–music mapping module.	Some routine training exercises; Individual session	Not clearly reported	Using device & PT	Music	1. Not clearly reported<br>2. An eight-track stereo instrumental ensemble containing melodic and percussive elements in a 4/4 time signature. These elements fulfill musical roles corresponding to percussion, melody, and harmony in a simplified pop music style, while allowing for real-time customization.<br>3. Computer-generated
Kantan (2023)	Auditory feedback on lower limb kinematics	Auditory sonification of swing phase kinematics using inertial sensors, generating ecological 'wading' sounds.	One-time test session, three feedback variants (fixed order: neutral, positive, negative), For each variant, 2 min length on average); Individual session	Rehabilitation clinics	PT	Sound	1. Researcher-selected<br>2. Sound intensity mapped to thigh/shank angular velocity during swing phase<br>3. Computer-generated
Kim (2014)	Rhythmic Auditory Stimulation (RAS) during arm reaching	Arm reaching task with/without RAS while seated	Single session; Individual session	Community center	OT	Sound	1. Researcher-selected<br>2. Metronome<br>3. Recorded
Ko (2016)	Smartphone-based RAS	Chronic stroke patients trained with a smartphone RAS app delivering various rhythmic tempos.	Single session trials at 5 tempos (-10, -5, baseline, +5, +10), for 10min each; Individual session	Rehabilitation center	PT	Sound	1. Participant-selected (preferred sound source from app options)<br>2. Seven possible rhythmic auditory stimuli<br>3. Recorded

Lee (2012)	Rhythmic Auditory Stimulation (RAS)	Participants walked under 5 different conditions:<br>(1) Normal walking without RAS (at comfortable speed)<br>(2) RAS matched to baseline speed (non-paretic leg)<br>(3) RAS 30% slower than baseline (non-paretic leg)<br>(4) RAS matched to baseline speed (paretic leg)<br>(5) RAS 30% faster than baseline (paretic leg)	One-time session,<br>5 gait trials: once 8 min (3min for warm-up, 5 min for gait training). There was an interval of 10 minutes between the trials; Individual session	Hospital	PT	Sound	1. Researcher-selected<br>2. Metronome beat (adjusted to each subject's cadence)<br>3. Recorded
Lee, S. Y. (2018)	Mental Singing session	Mental singing while walking intervention:<br>7 consecutive tasks: (1) listening to the song; (2) clapping hands or tapping toes on the ground while listening to the song; (3) singing; (4) clapping or tapping while singing; (5) singing while sitting on a chair and walking in place; (6) walking while singing, and (7) walking while mentally singing the song	One-time session; Individual session	Hospital	Not clearly reported	Music	1. Researcher-selected<br>2. A well-known Korean children's song 'Santokki (a jack rabbit)'<br>3. Recorded
Mizuta (2022)	Rhythmic auditory cueing (RAC)	The participants were instructed to walk in the RAC conditions. They were asked to match the heel contact timing to the beat of the metronome.	One-time trial session, walking 2-3 times on a 10m walkway with rhythmic auditory cueing;<br>Individual session	Hospital	PT	Sound	1. Researcher-selected<br>2. Metronome beat. The tempo was determined by calculating the cadence of comfortable walking before starting the measurement.<br>3. Recorded
Pelton (2010)	Hemiparetic treadmill walking with metronome synchronizati on and unpredictable	Treadmill walking synchronized with metronome pulses<br>*Phases: Baseline trials without phase shifts followed by trials with unpredictable phase shifts	One-time session,<br>during five 100-pulse trials, a fixed-phase baseline was followed; Individual session	Community-dwelling adults	Researcher	Sound	1. Researcher-selected<br>2. Audible metronome beeps<br>3. Computer-generated

	phase shifts						
Peyre (2020)	Treadmill locomotion	Arrhythmic music listening during treadmill locomotion at the preferred speed of each individual<br>Without sound, with a melody and with aleatory electronic sound	One-time trial session, participants walked on the treadmill for 5-10 min before recordings and walked for 2-4 min with stability; Individual session	Hospital	Researcher	Music & sound	1. Researcher-selected<br>2. Music with irregular tempo: pleasant melody vs. unpleasant sound (i.e., aleatory electronic sounds: AES)<br>3. Recorded
Prassas (1997)	Rhythmic auditory cueing (RAC)	Gait trials with metronome cuing vs. baseline	One-time session; Individual session	Rehabilitation center	PT	Sound	1. Researcher-selected<br>2. Metronome<br>3. Recorded
Roerdink (2007)	Rhythmic Auditory Cueing (RAC)	Walking trials with and without auditory cueing (metronome)	One-time session; Individual session	University clinical laboratory	Researcher	Sound	1. Researcher-selected<br>2. Isochronous beats<br>3. Recorded
Roerdink (2009)	Acoustically Paced Treadmill Walking with Rhythmic Auditory Cueing(RAC)	Treadmill walking at preferred speed and cadence under no metronome, single-metronome (pacing only paretic or nonparetic steps), and double-metronome (pacing both footfalls) conditions	Three sessions, performed on separate days (approximately 2 hours including rest per session.);<br>Individual session	Rehabilitation center	Device	Sound	1. Researcher-selected<br>2. Metronome beat<br>3. Recorded
Secoli (2011)	A tracking task (similar to commonly-used-robotic therapy)	Tracking a visual target with their affected arms while receiving adaptive assistance from a robotic arm exoskeleton with auditory feedback;<br>A tracking task in 4 conditions (1) the baseline tracking task alone; (2) tracking while also performing a visual distracter task; (3) tracking with the visual distracter and sound feedback; and (4) tracking with sound feedback.	One-time session; Individual session	Clinic	Device	Sound	1. Researcher-selected<br>2. A sequence of tonal beeps (800 Hz)<br>3. Computer-generated

Sethi (2017)	Upper extremity movements	Participants performed reach-to-point movements (using the index finger between target marks) under three conditions: (1) self-paced reaching at preferred speed, (2) reaching as fast as possible, (3) reaching with rhythmic auditory cues generated by a metronome matched to each pts' preferred speed. (30 trials)	One-time test session, patients performed four trials in each condition; Individual session	Research center	Not clearly reported	Sound	1. Researcher-selected<br>2. Metronome beat<br>3. Recorded
Thaut (1993)	Rhythmic Auditory Cuing (RAC)	(1) The gait cadence was calculated for each subject during baseline walk. (2) The tempo of the music was set to each subject's gait cadence. (3) The music was turned on and the subjects were asked to tap their feet and/or hands to the music for one minute. (4) After that, the subjects were asked to walk again and step to the accentuated beats of the music as closely as possible.	Three times over a five-week period. Testing sessions were scheduled one week apart; Individual session	Hospital	Not clearly reported	Music	1. Researcher-selected<br>2. The rhythm stimulus was an original musical composition in renaissance dance style, written in 4/4 time signature and orchestrated for woodwinds, harpsichord, and percussion. The first and third beats in each measure were accentuated by a tambourine beat to enhance the rhythmic "feeling" of the music.<br>3. Recorded
Thaut (2002)	Rhythmic metronome cuing for reaching movements	Patients were asked to move their arm in time with the rhythm by touching the sensors on the beat. The frequency of the rhythmic cue was matched to the patient's self-paced movement frequency which was assessed before the start of the trial.	One-time trial session; Individual session	Research center or therapy center	Not clearly reported	Sound	1. Researcher-selected<br>2. The auditory rhythm consisted of a metronome-like 1000 Hz square wave tone with a 50 ms plateau time produced by a computerized MIDI-sequencing sound software (Logic 2.5)<br>3. Recorded
Wright (2016)	Metronome cueing of gait	Walk under two different conditions;<br>(1) standard walking trials<br>(2) walking trials with metronome cueing	One-time session, Three standard gait trials and three gait trials with an auditory metronome; Individual session	Not clearly reported	Not clearly reported	Sound	1. Researcher-selected<br>2. Metronome beat<br>3. Recorded

Note. MT = music therapist; OT = occupational therapist; PT = physical therapist.
