# Supplementary material for: Reconsidering music in stroke rehabilitation: a scoping review from auditory stimulus to relational process
Source: Front Psychol. 2026 Jun 5;17:1774971. doi: 10.3389/fpsyg.2026.1774971 (PMC13279217; doi:10.3389/fpsyg.2026.1774971)
Supplement: SUPPLEMENTARY Table S4 — Full database-specific search strategies, including Boolean operators, search strings, and applied filters for all seven databases. [file Data_sheet_4.pdf]

Supplementary Material Table S4. Full database-specific search strategies

Search terms were organized into three conceptual blocks and adapted to the syntax of each database. Truncation was applied using the asterisk (*) where supported. Searches were limited to English-language records, with no restriction on publication date.

Conceptual block	Keywords
Population	stroke OR CVA
Music/sound or auditory stimulation	music* OR play* OR sing* OR listen* OR sound* OR rhythm* OR melod*
Rehabilitation, motor function, and delivery context	rehabilitat* OR motor* OR physical* OR lower OR upper OR gait OR intervention* OR therap* OR therapeutics OR home OR remote OR tele*

Database	Search field/syntax	Full search string	Limits/filters
PubMed	[Title/Abstract]	((stroke[Title/Abstract] OR CVA[Title/Abstract]) AND (music*[Title/Abstract] OR play*[Title/Abstract] OR sing*[Title/Abstract] OR listen*[Title/Abstract] OR sound*[Title/Abstract] OR rhythm*[Title/Abstract] OR melod*[Title/Abstract]) AND (rehabilitat*[Title/Abstract] OR motor*[Title/Abstract] OR physical*[Title/Abstract] OR lower[Title/Abstract] OR upper[Title/Abstract] OR gait[Title/Abstract] OR intervention*[Title/Abstract] OR therap*[Title/Abstract] OR therapeutics[Title/Abstract] OR home[Title/Abstract] OR remote[Title/Abstract] OR tele*[Title/Abstract]))	English language; no publication date restriction
CINAHL (EBSCOhost)	TX = all text	TX (stroke OR CVA) AND TX (music* OR play* OR sing* OR listen* OR sound* OR rhythm* OR melod*) AND TX (rehabilitat* OR motor* OR physical* OR lower OR upper OR gait OR intervention* OR therap* OR therapeutics OR home OR remote OR tele*)	English language; no publication date restriction
Scopus	TITLE-ABS-KEY	TITLE-ABS-KEY (stroke OR CVA) AND TITLE-ABS-KEY (music* OR play* OR sing* OR listen* OR sound* OR rhythm* OR melod*) AND TITLE-ABS-KEY (rehabilitat* OR motor* OR physical* OR lower OR upper OR gait OR intervention* OR therap* OR therapeutics OR home OR remote OR tele*)	English language; no publication date restriction
Cochrane Library	:ti,ab,kw	(stroke OR CVA):ti,ab,kw AND (music* OR play* OR sing* OR listen* OR sound* OR rhythm* OR melod*):ti,ab,kw AND (rehabilitat* OR motor* OR physical* OR lower OR upper OR gait OR intervention* OR therap* OR therapeutics OR home OR remote OR tele*):ti,ab,kw	English language where available; no publication date restriction

Database	Search field/syntax	Full search string	Limits/filters
PsycINFO (via ProQuest)	NOFT = citation/abstract fields excluding full text	NOFT(stroke OR CVA) AND NOFT(music* OR play* OR sing* OR listen* OR sound* OR rhythm* OR melod*) AND NOFT(rehabilitat* OR motor* OR physical* OR lower OR upper OR gait OR intervention* OR therap* OR therapeutics OR home OR remote OR tele*)	English language; no publication date restriction
ProQuest	NOFT = citation/abstract fields excluding full text	NOFT(stroke OR CVA) AND NOFT(music* OR play* OR sing* OR listen* OR sound* OR rhythm* OR melod*) AND NOFT(rehabilitat* OR motor* OR physical* OR lower OR upper OR gait OR intervention* OR therap* OR therapeutics OR home OR remote OR tele*)	English language; no publication date restriction
Web of Science	TS = topic	TS=(stroke OR CVA) AND TS=(music* OR play* OR sing* OR listen* OR sound* OR rhythm* OR melod*) AND TS=(rehabilitat* OR motor* OR physical* OR lower OR upper OR gait OR intervention* OR therap* OR therapeutics OR home OR remote OR tele*)	English language; no publication date restriction

Note. CVA = cerebrovascular accident. The asterisk (*) indicates truncation. The search strategy was adapted to each database according to its supported field tags and search operators.
