# Supplementary material for: Reconsidering music in stroke rehabilitation: a scoping review from auditory stimulus to relational process
Source: Front Psychol. 2026 Jun 5;17:1774971. doi: 10.3389/fpsyg.2026.1774971 (PMC13279217; doi:10.3389/fpsyg.2026.1774971)
Supplement: SUPPLEMENTARY File 6 — Completed PRISMA-ScR checklist. [file Data_sheet_6.pdf]

Supplementary Material 6. PRISMA-ScR Checklist

Preferred Reporting Items for Systematic reviews and Meta-Analyses extension for Scoping Reviews

Note. Page numbers refer to the revised manuscript version rendered for review. Supplementary materials are listed according to the numbering used in the revised submission.

Section	Item	No.	PRISMA-ScR checklist item	Reported on page/section
TITLE	Title	1	Identify the report as a scoping review.	p. 1
ABSTRACT	Structured summary	2	Provide a structured summary that includes, as applicable: background, objectives, eligibility criteria, sources of evidence, charting methods, results, and conclusions that relate to the review questions and objectives.	p. 1
INTRODUCTION	Rationale	3	Describe the rationale for the review in the context of what is already known. Explain why the review questions/objectives lend themselves to a scoping review approach.	pp. 1-3
INTRODUCTION	Objectives	4	Provide an explicit statement of the questions and objectives being addressed with reference to their key elements (e.g., population or participants, concepts, and context) or other relevant key elements used to conceptualize the review questions and/or objectives.	p. 3
METHODS	Protocol and registration	5	Indicate whether a review protocol exists; state if and where it can be accessed (e.g., a Web address); and if available, provide registration information, including the registration number.	p. 3
METHODS	Eligibility criteria	6	Specify characteristics of the sources of evidence used as eligibility criteria (e.g., years considered, language, and publication status), and provide a rationale.	p. 4
METHODS	Information sources	7	Describe all information sources in the search (e.g., databases with dates of coverage and contact with authors to identify additional sources), as well as the date the most recent search was executed.	pp. 3-4; Supplementary Material 4
METHODS	Search	8	Present the full electronic search strategy for at least 1 database, including any limits used, such that it could be repeated.	pp. 3-4; Supplementary Material 4
METHODS	Selection of sources of evidence	9	State the process for selecting sources of evidence (i.e., screening and eligibility) included in the scoping review.	p. 4
METHODS	Data charting process	10	Describe the methods of charting data from the included sources of evidence (e.g., calibrated forms or forms that have been tested by the team before their use, and whether data charting was done independently or in duplicate) and any processes for obtaining and confirming data from investigators.	pp. 4-5; Supplementary Material 5
METHODS	Data items	11	List and define all variables for which data were sought and any assumptions and simplifications made.	pp. 4-5; Supplementary Material 5
METHODS	Critical appraisal of individual sources of evidence	12	If done, provide a rationale for conducting a critical appraisal of included sources of evidence; describe the methods used and how this information was used in any data synthesis, if appropriate.	p. 5
METHODS	Synthesis of results	13	Describe the methods of handling and summarizing the data that were charted.	p. 5
RESULTS	Selection of sources of evidence	14	Give numbers of sources of evidence screened, assessed for eligibility, and included in the review, with reasons for exclusions at each stage, ideally using a flow diagram.	p. 5; Figure 1
RESULTS	Characteristics of sources of evidence	15	For each source of evidence, present characteristics for which data were charted and provide the citations.	pp. 5-6; Supplementary Material 1
RESULTS	Critical appraisal within sources of evidence	16	If done, present data on critical appraisal of included sources of evidence (see item 12).	Not applicable; see p. 5
RESULTS	Results of individual sources of evidence	17	For each included source of evidence, present the relevant data that were charted that relate to the review questions and objectives.	pp. 5-8; Supplementary Materials 1-3
RESULTS	Synthesis of results	18	Summarize and/or present the charting results as they relate to the review questions and objectives.	pp. 5-8; Supplementary Materials 1-3

Section	Item	No.	PRISMA-ScR checklist item	Reported on page/section
DISCUSSION	Summary of evidence	19	Summarize the main results, including an overview of concepts, themes, and types of evidence available; link to the review questions and objectives; and consider the relevance to key groups.	pp. 8-10
DISCUSSION	Limitations	20	Discuss the limitations of the scoping review process.	p. 10
DISCUSSION	Conclusions	21	Provide a general interpretation of the results with respect to the review questions and objectives, as well as potential implications and/or next steps.	p. 10
FUNDING	Funding	22	Describe sources of funding for the included sources of evidence, as well as sources of funding for the scoping review. Describe the role of the funders of the scoping review.	p. 11

Abbreviations. JBI = Joanna Briggs Institute; PRISMA-ScR = Preferred Reporting Items for Systematic reviews and Meta-Analyses extension for Scoping Reviews.

Source. Adapted from Tricco AC, Lillie E, Zarin W, et al. PRISMA Extension for Scoping Reviews (PRISMA-ScR): Checklist and Explanation. Annals of Internal Medicine. 2018;169(7):467-473. doi:10.7326/M18-0850
